# HSP90 acts as a senomorphic target in senescent retinal pigmental epithelial cells

**DOI:** 10.18632/aging.203496

**Published:** 2021-09-08

**Authors:** Dan-Dan Chen, Xuyan Peng, Yuxuan Wang, Mingjun Jiang, Mengjiao Xue, Guohui Shang, Xuhui Liu, Xiaolin Jia, Baixue Liu, Yingwei Lu, Hongmei Mu, Fengyan Zhang, Yanzhong Hu

**Affiliations:** 1 The Division of Ophthalmology and Vision Science, Department of Ophthalmology, The First Affiliated Hospital of Zhengzhou University, Zhengzhou University, Zhengzhou, China; 2 The jointed National Laboratory of Antibody Drug Engineering, Department of Cell Biology and Genetics, The College of Basic Medical Science of Henan University, Kaifeng, China; 3 Kaifeng Key laboratory of Cataracts and Myopia, Eye Disease Institute, Kaifeng Central Hospital, Kaifeng, China; 4 Department of Medical Genetics and Cell Biology, School of Basic Medical Sciences, Zhengzhou University, Henan 450001, China

**Keywords:** HSP90, senotheray, NF-kb, HIF1α, β-galactosidase

## Abstract

The senescence of retinal pigment epithelial (RPE) cells is associated with age-related macular degeneration (AMD), a leading cause of blindness in the world. HSP90 is a predominant chaperone that regulates cellular homeostasis under divergent physio-pathological conditions including senescence. However, the role of HSP90 in senescent RPE cells still remains unclear. Here, we reported that HSP90 acts as a senomorphic target of senescent RPE cells *in vitro*. Using H_2_O_2_-induced senescent ARPE-19 cells and replicative senescent primary RPE cells from rhesus monkey, we found that HSP90 upregulates the expression of IKKα, and HIF1α in senescent ARPE-19 cells and subsequently controls the induction of distinct senescence-associated inflammatory factors. Senescent ARPE-19 cells are more resistant to the cytotoxic HSP90 inhibitor IPI504 (IC50 = 36.78 μM) when compared to normal ARPE-19 cells (IC50 = 6.16 μM). Administration of IPI504 at 0.5–5 μM can significantly inhibit the induction of IL-1β, IL-6, IL-8, MCP-1 and VEGFA in senescent ARPE-19 and the senescence-mediated migration of retinal capillary endothelial cells *in vitro*. In addition, we found that inhibition of HSP90 by IPI504 reduces SA-β-Gal’s protein expression and enzyme activity in a dose-dependent manner. HSP90 interacts with and regulates SA-β-Gal protein stabilization in senescent ARPE-19 cells. Taken together, these results suggest that HSP90 regulates the SASP and SA-β-Gal activity in senescent RPE cells through associating with distinctive mechanism including NF-κB, HIF1α and lysosomal SA-β-Gal. HSP90 inhibitors (e.g. IPI504) could be a promising senomorphic drug candidate for AMD intervention.

## INTRODUCTION

Age-related macular degeneration (AMD) affects approximately 0.4–8.7% of people over the age of 50 [1] and is a leading cause of blindness among the elderly in the developed world [2]. The majority of affected individuals (about 80–85%) suffer from dry AMD, characterized by atrophy of photoreceptor cells, ganglia cells, and retinal pigment epithelial cells (RPE), inflammation, drusen accumulation, and thickening of Bruch’s membrane, while about 20% of patients have wet AMD, which is characterized by retinal neovascularization and edema. An increase in senescent cell numbers in RPE is associated with early onset AMD. Targeting senescent cells to undergo apoptosis (senolytics) or inhibition of senescence-associated secretion phenotype (senomorphics) can prevent or improve aging and age-associated diseases (e.g., atherosclerosis, arthritis, chronic fibrosis and cancer metastasis and therapeutic resistance) [3], and p16INK4, Bcl-2 and HSP90 have been reported to act as senotherapeutic targets [3, 4]. Activation of RPE cell regeneration or transplantation of RPE cells that were differentiated from autologous inducible pluripotential stem cells (iPSC) improved vision acutely in mouse AMD models [5, 6], and the transplantation strategy using hECS-derived RPE sheet is currently in clinic trial [7–9]. Therefore, senotherapy of senescent RPE is a potential and promising strategy for AMD intervention.

The retinal pigment epithelium (RPE) is a monolayer of polar pigmented epithelial cells located between the photoreceptors and Bruch’s membrane. RPE is essential for the maintenance and survival of overlying photoreceptor cells as it performs a number of critical functions, such as formation of the outer blood retinal barrier, transepithelial transport, maintenance of the retinoid cycle, phagocytosis, degradation of photoreceptor outer segment tips, and protection against light and oxidative stress [10]. RPE cells undergo senescence with aging [11, 12]. An accumulation of senescent cells is observed in senile retina, especially in AMD patients [13]. Aging and age-associated stresses such as blue light, lipofuscin, smoking, glaucoma, chronic inflammation and other systemic diseases, which induce ROS levels in RPE cells, trigger RPE cells to undergo senescence by activating the p53-p21 or p16INK4-pRB pathways [14]. Senescent RPE was also observed in the AMD-like retina model generated by genetic mutations in ApoE, RPE65, Lamp-2, and MCP-1 [15–17]. RPE cells that have undergone senescence lose their function, such as the ability to degrade the outer segments of photoreceptors, resulting in accumulation and deposition of lipofuscin extracellularly, forming drusen [12]. The intracellular accumulation of lipofuscin triggers senescence-associated mitochondrial dysfunction, which in turn activates the signal pathways (such as NF-κB and HIF1α) to upregulate the senescence associated secretion phenotype (SASP) increasing the expression of IL-1β, IL-6, IL-8, MCP-1 or growth factors like TGF-beta and VEGF, which then leads to divergent pathological changes in the retina, (i.e., chronic inflammation, retinal neurons apoptosis or senescence and neovascularization of choroid) [18, 19]. Senescent RPE cells can survive for a period of time before they are cleared by their neighboring healthy RPE or macrophages via phagocytosis [20]. The survival of senescent cells is associated with an upregulation of pro-survival factors such as Bcl-2, Bcl-XL and/or heat shock proteins [21]. Some of these factors (Bcl-2 and Bcl-xl) act as senolytic targets in some specific tissues such as hair follicle, skeletal muscle, and hematopoietic stem cells [22]. However, investigations are ongoing to assess whether these pro-survival factors are appropriated for senotherapy of senescent RPE cells [23].

Heat shock protein 90 is an ATP-dependent chaperon that is conserved from bacteria to human. In mammals, there are two types of HSP90 proteins, HSP90α (referenced as HSP90 in text) and HSP90β, which share 85% homology to HSP90α. The two isoforms are encoded by two distinct genes, HSP90AA and HSP90AB [24]. The expression of HSP90α is regulated in a stress-dependent manner. Heat shock factor 1 (HSF1) is the predominant upstream transcription factor that controls the expression of HSP90α in response to stresses [25]. In contrast, HSP90β is constitutively expressed in most of tissues. HSP90 proteins are abundant in the cells, accounting for roughly 2% of total proteins [26]. HSP90 utilizes its ATPase activity to assist in protein folding or degradation and to regulate cellular proteostasis in response to divergent physio-pathological stresses [24]. HSP90 is involved in regulating a myriad of pathophysiologic processes (i.e., cell proliferation and differentiation, wound healing, inflammation, tumor cell proliferation, metastasis and chemotherapy resistance, programmed cellular apoptosis and senescence) through chaperoning divergent signaling factors (e.g., EGFR, TGFR, CDC37, AKT and NF-κB pathways) [24]. In *C. elegans*, the HSP90 inhibitor 17-AAG can extend the lifespan of *C. elegans* by activating HSF1-mediated heat shock response [27]. In mouse models, 17-AAG prolongs the lifespan of ERRC1-deficient progeroid mice [28], and can improve recognition in Alzheimer disease models by reducing amyloid plague formation and tau hyperphosphorylation [29]. Inhibition of HSP90 by geldanamycin improves the vision of rhodopsin/P23H transgenic mice, mainly by activating the HSF1-mediated heat shock response. This suggests that HSP90 could function as a senotherapeutic target. However, On the other hand, HSP90 has also been reported to act as an anti-aging regulator. Deletion of HSP90β induces skeletal muscle cells to undergo senescence by inactivating MDM2 [30]. The inhibition of HSP90 by its inhibitor 17-AAG promotes human lung cancer cells to undergo senescence *in vitro* through stabilization of the p14^arf^ protein. HSP90-CHIP was found to regulate p14^arf^ protein degradation in lysosomes in a lamp2-mediated manner, and this make P14^arf1^ positive small lung cancer cells sensitive to HSP90 inhibitor [31]. These divergent results suggested that HSP90 play a complex role in the regulation of programmed senescence.

HSP90 is induced to protect RPE cells from oxidative stress [32]. The inhibition of HSP90 attenuates the proliferation of ARPE-19 cells by stopping the cell cycle at the G1/S phase *in vitro* [33]. HSP90 regulates the expression and the secretion of VEGFA under hypoxic conditions [34]. HSP90 regulates the expression and secretion of inflammatory cytokines (such as IL-1β) under IL-1α priming by regulating the degradation of NLRP3 proteins in the autophagy-lysosome pathway or NF-κB pathway [35]. However, the regulation of HSP90 in senescent RPE cells has yet to be studied.

In this paper, we investigated the regulation of HSP90 on the cellular homeostasis of senescent RPE cells *in vitro* and aim to determine whether HSP90 could act as a senotherapeutic target. We found that inhibition of HSP90 by its inhibitor IPI-504 suppressed the mRNA expression and secretion of senescence-associated inflammatory factors without inducing apoptosis in senescent RPE cells *in vitro*, and attenuated SASP-mediated human endothelial cell migration. HSP90 upregulated the expression of senescence-associated inflammatory factors via the NF-κB pathway and HIF1α pathways. In addition, HSP90 interacts with the SA-β-Gal protein, and inhibition of HSP90 by IPI-504 reduces SA-β-Gal protein expression and enzyme activity in senescent ARPE-19 cells. Our results suggest that HSP90 is a potential senomorphic candidate for AMD intervention.

## RESULTS

### Short-time treatment of H_2_O_2_ induces RPE cells to undergo senescence *in vitro*

**Figure 1:**
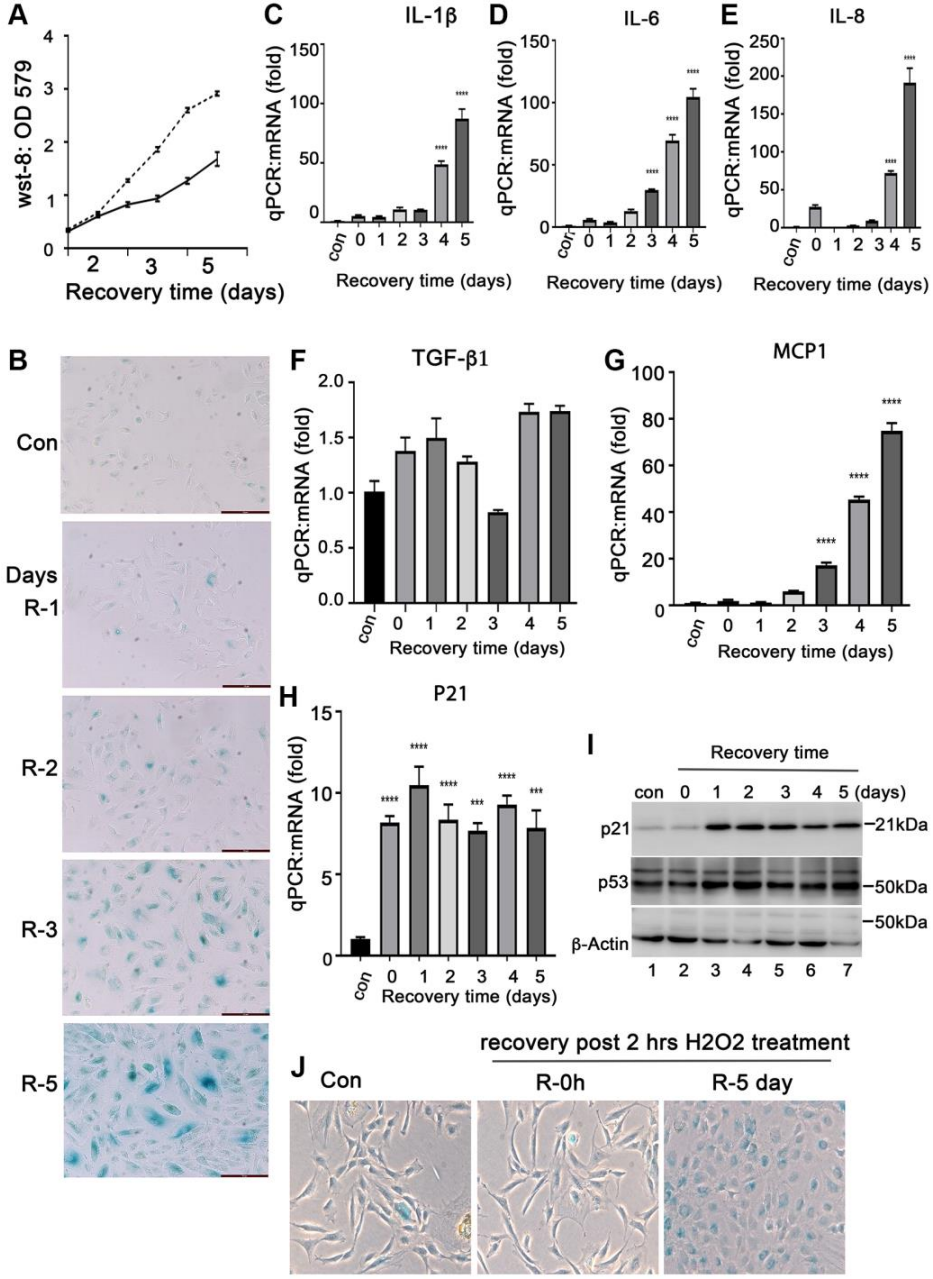
**Induction of senescent ARPE-19 cells by H_2_O_2_*in vitro*.** (**A**) Proliferation of ARPE-19 during recovery after 2 hours of H_2_O_2_ treatment using the CCK-8 assay (WST-8, 2-methoxy-4-nitrophenyl)-3- (4-nitrophenyl)-5- (2, 4-disulfophenyl)-2H-tetrazolium). (**B**) Staining for SA-β-Gal activity in ARPE-19 cells treated with 200 μM H_2_O_2_ for 2 hours followed by recovery for 1, 2, 3, 4 and 5 days. (**C**–**H**) Quantitative PCR to detect mRNA expression of IL-1β, IL-6, IL-8, MCP-1, TGF-β1 and P21 in ARPE-19 cells treated with H_2_O_2_ in the same way as in B. (**I**) Immunoblot of P53 and p21 protein expression in ARPE-19 cells treated with H_2_O_2_ in the same way as in B. (**J**) Detection of SA-β-Gal activity in passage 3 primary monkey RPE cells treated with H_2_O_2_ for two hours followed by recovery in normal media for 0 and 5 days. The two-tailed unpaired *t*-test was used for statistical analysis. The data were collected from three independent experiments (*n* = 3).

**Figure 2:**
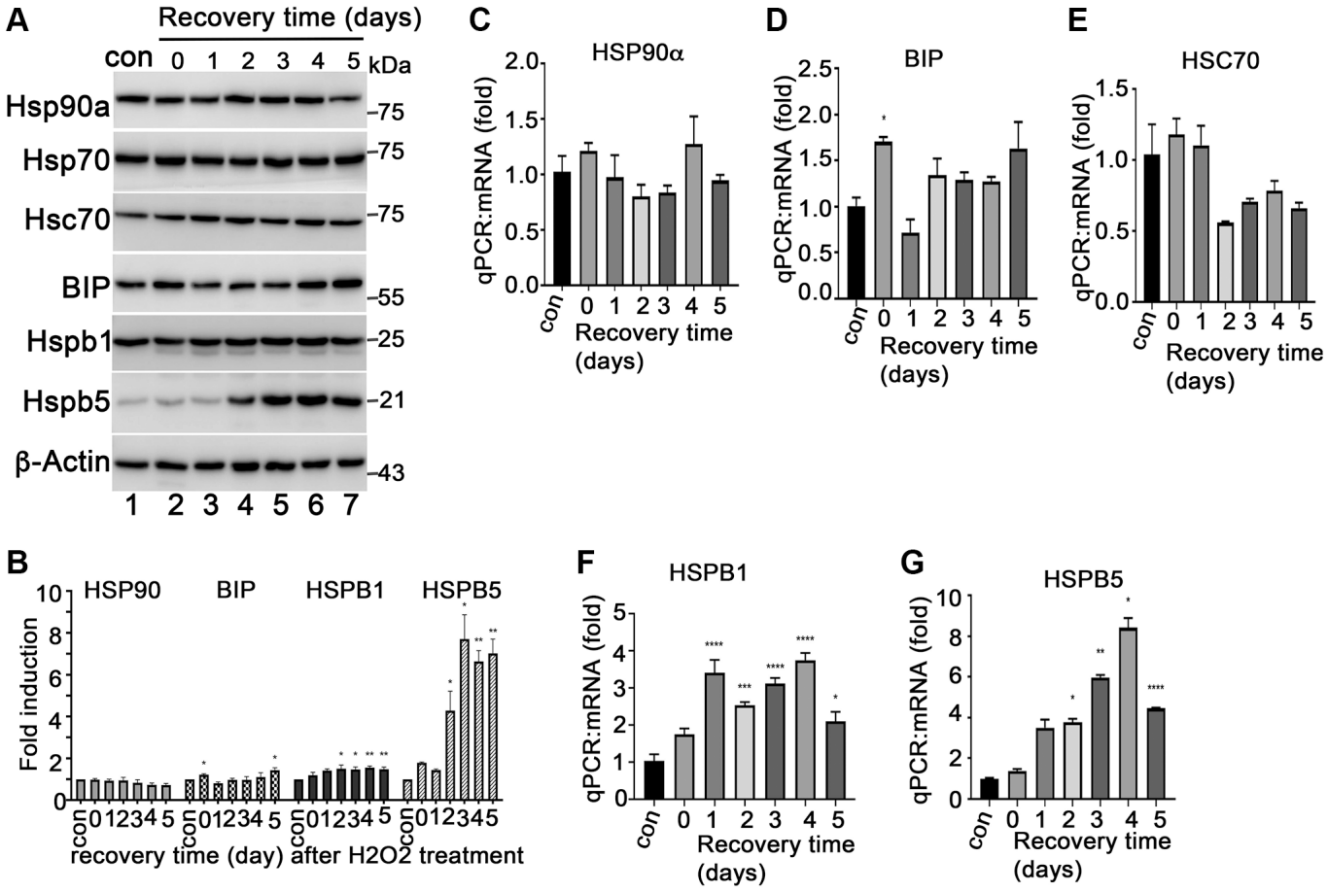
**Expression of heat shock proteins during ARPE-19 senescence.** (**A**) Immunoblot of HSP90α, HSP70, HSC70, BIP, HSPB1 (HSP27), HSPB5 (αB-crystallin) and β-actin in ARPE-19 cells treated with H_2_O_2_ in the same way as Figure 1B. (**B**) Densitometry quantitation of protein bands in A in image J. The data shown are mean ± SD. The two-tailed unpaired *t*-test was used for statistical analysis (*n* = 3). (**C**–**G**) Quantitative PCR to measure the expression of HSP90α, BIP, HSC70, HSPB1 and HSPB5 in the cells treated with H_2_O_2_ the same way as A. The data were from three independent experiments. The two-tailed unpaired *t*-test was used for statistical analysis (*n* = 3).

Senescent RPE cells is closely associated with AMD development. Oxidative stress is a common factor causing RPE cell senescence. To study the characteristics of senescent RPE cells, we established a model of senescent RPE cells by treating ARPE-19 cells with H_2_O_2_ *in vitro*. The ARPE-19 cells were treated with H_2_O_2_ at 50, 200, 350 and 500 μM for 2 hours followed by recovery for 72 hours in normal media. We found that 200–300 μM of H_2_O_2_ are proper doses to induce ARPE-19 cells to undergo senescence (Supplementary Figure 1). The increase H_2_O_2_ caused ARPE-19 cell death (data not shown). Compared to normal ARPE-19 cells, the short term treatment with 200 μM of H_2_O_2_ attenuated ARPE-19 cells growth (Figure 1A), and the cells continued to survive at least for 10 days after treatment (data not shown). We examined the senescent biomarkers of these cells. SA-β-Gal positive cells were detectable in day 2 after H_2_0_2_ treatment, and the number of positive cells continued to increase with prolonged recovery time after treatment (Figure 1B) with the increases of the expression of senescence-associated cytokines, such as IL-1β, IL-6, IL-8, MCP-1, but not TGF-β1 (Figure 1C–1G) and other senescence markers, such as P21 and P53 at both mRNA and protein levels (Figure 1H and 1I). P16INK4 was not detected at both mRNA and protein levels (data not shown), and the reason for this is still under investigation. Interestingly, we found that the expression of anti-apoptotic proteins such as Bcl-xL (Supplementary Figure 2A and 2B), c-Flip (Supplementary Figure 2C) and aB-crystallin (Figure 2G) were induced in the ARPE-19 cells that underwent senescence, which may explain the ability of senescent RPE to survive. In addition, we also examined this H_2_O_2_-induced senescence model on monkey primary RPE cells. We treated the primary monkey RPE cells at passage 3 with 250 μM H_2_O_2_ for 2 hours followed by recovery for 0 and 4 days in complete media. Most of day-4 recovery cells were SA-β-Gal positive compared to the cells cultured in control conditions or in H_2_O_2_-treated cells without recovery (Figure 1J). Taken together, these results indicated that treatment of H_2_O_2_ at a concentration of 200 uM–300 uM for 2 hours could induce RPE cells to undergo cellular senescence *in vitro*. Most RPE cells undergo senescence at day 3 recovery post- H_2_O_2_ treatment. These senescent cells could survive for a period of time.

### Inhibition of HSP90 suppresses the SASP in senescent RPE cells *in vitro*

**Figure 3:**
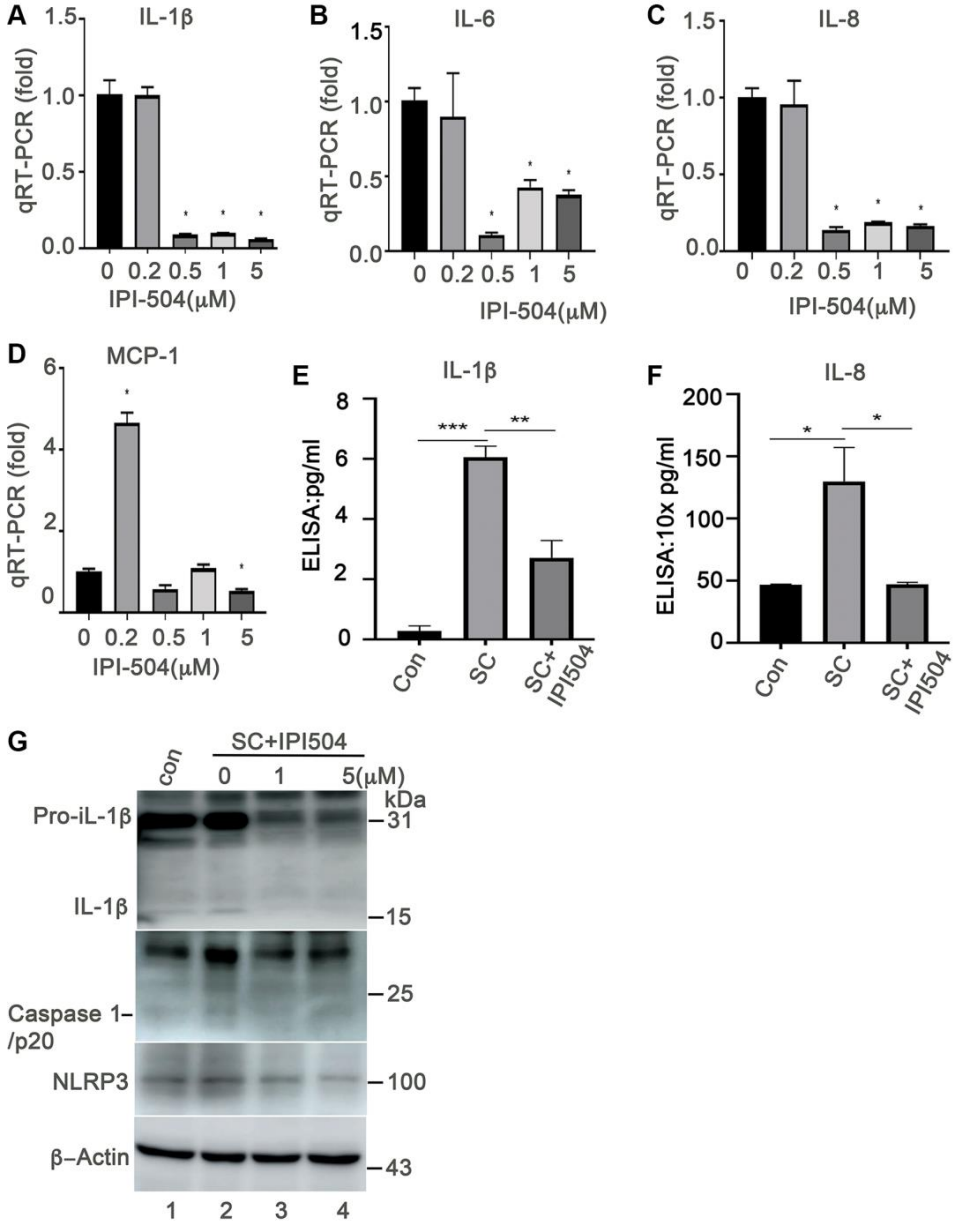
**Heat shock protein 90 inhibitor IPI-504 suppresses the expression and secretion of senescence-associated inflammatory factors in senescent ARPE-19. (A**–**D**) quantitative PCR measuring the expression of IL-1β, IL-6, IL-8, MCP-1 in day-4 senescent ARPE cells treated with media containing PBS (0) or IPI-504 at the concentrations of 0.2, 0.5, 1 and 5 μM. (**E**–**F**) ELISA assay measuring the secretion of IL-1β and IL-8 in the supernatants of senescent ARPE-19 cells treated with IPI-504. con: proliferative ARPE-19 cells, SC: senescent ARPE-19 cells (day-4), SC+IPI504: Day-4 SC cells were treated with 1 μM IPI-504 for 24 hours. (**G**) Immunoblot the expression of IL-1β, cleaved caspase 1, NLRP3 and GAPDH in control ARPE-19 cells (lane 1), or day-4 senescent ARPE-19 cells treated with IPI-504 at 0, 1 and 5 μM (lanes 2, 3 and 4). The data in each quantitation figure were collected from four independent experiments (*n* = 4). The two-tailed unpaired *t*-test was used for statistical analysis, ^*^p < 0.05, ^***^p < 0.001.

HSP90 plays multiple functions in orchestrating the proteostasis of cells under different conditions. Inhibition of HSP90 reduces the expression of senescence-associated inflammatory factors in senescent cancer cells [36] and extends the lifespan of Ercc1-defficient progeroid mice by inducing senescence cell apoptosis [28]. These results revealed the HSP90 might act as a novel senotherapeutic target. We tested the expression profile of heat shock proteins, including HSP90, in the H_2_O_2_-induced senescent ARPE-19 cells. The results showed that HSP90 was abundantly and constantly expressed at both mRNA and protein levels in control and senescent ARPE-19 cells with no difference between the treatment groups (Figure 2A–2C). ER stress-associated BIP and small heat shock proteins (e.g., Hsp25 and αB-crystallin) were upregulated in the H_2_O_2_ induced senescent cells compared to that in normal ARPE-19 cells (Figure 2A, 2D, 2F and 2G). However, Hsc70 and HSP70 were not changed in senescent cells (Figure 2A and 2E). The results suggested that heat shock proteins were reprogrammed during senescence of RPE cells. To determine, whether HSP90 is essential for senescent RPE cell homeostasis, we further treated senescent ARPE-19 cells with IPI-504, an inhibitor of HSP90 that binds to and inactivates HSP90’s ATPase activity. IPI-504 exhibited more cytotoxicity to the proliferative ARPE-19 cells (IC50 = 6.16 μM, R2 = 0.93) than to the senescent ARPE-19 cells (IC50 = 36.78 μM; R2 = 0.99) (Supplementary Figure 3) *in vitro*. We treated day-4 senescent ARPE-19 cells with IPI-504 at concentrations of 0, 0.2, 0.5, 1 and 5 μM for 24 h. No apoptosis was observed in the senescent ARPE-19 cells treated with IPI-504 at those concentrations (Supplementary Figure 2B and data not shown). We further measured the expression of senescence-associated inflammatory factors with qPCR. As the results indicated in Figure 3, IPI-504 inhibited the mRNA expression of IL-1β, IL-6, IL-8, and MCP-1 in a dose dependent manner (Figure 3A–3D), and the tested pre-IL-1β protein expression (Figure 3G). As expected, the results of the ELISA indicated that IPI-504 also reduced the amount of IL-1β and IL-8 proteins in the supernatants of senescent ARPE-19 cells (Figure 3E and 3F). Hsp90 was reported to regulates IL-1β secretion by associating with inflammasome including NLRP3 and caspase 1 [35]. The data in Figure 3G showed that the pre-IL-β and NLRP3 proteins were upregulated in senescent ARPE-19 cells compared to normal ARPE-19 cells (Figure 3G lanes 1 and 2). IPI-504 reduced the expression of NLRP3 protein, cleaved caspase 1 and matured IL-1β in senescent ARPE-19 cells (Figure 3G, lanes 2–4). Therefore, these results suggested IPI-504 inhibited not only pre-IL-1β protein expression but also its maturation for secretion.

In addition, we also tested this inhibitory effect of IPI-504 on the primary monkey RPE cells that underwent replicative senescent *in vitro*. The primary monkey RPE cells underwent senescence after 8 passages (Supplementary Figure 4A). IPI-504 was then added to the senescent cells for 24 hours. The qPCR results indicated that IPI-504 down-regulated the mRNA expression of IL-1β, IL-6, IL-8, MCP-1 and TGF-β (Supplementary Figure 4B–4F), which is consistent with the results in Figure 3. Taken together, these results suggested that IPI-504 exhibited an inhibitory effect on the expression and secretion of senescence-associated cytokines in both stress-induced and replicative senescent RPE cells *in vitro*.

### HSP90 upregulates the SASPs by associating with NF-κB pathways in senescent ARPE-19 cells

**Figure 4:**
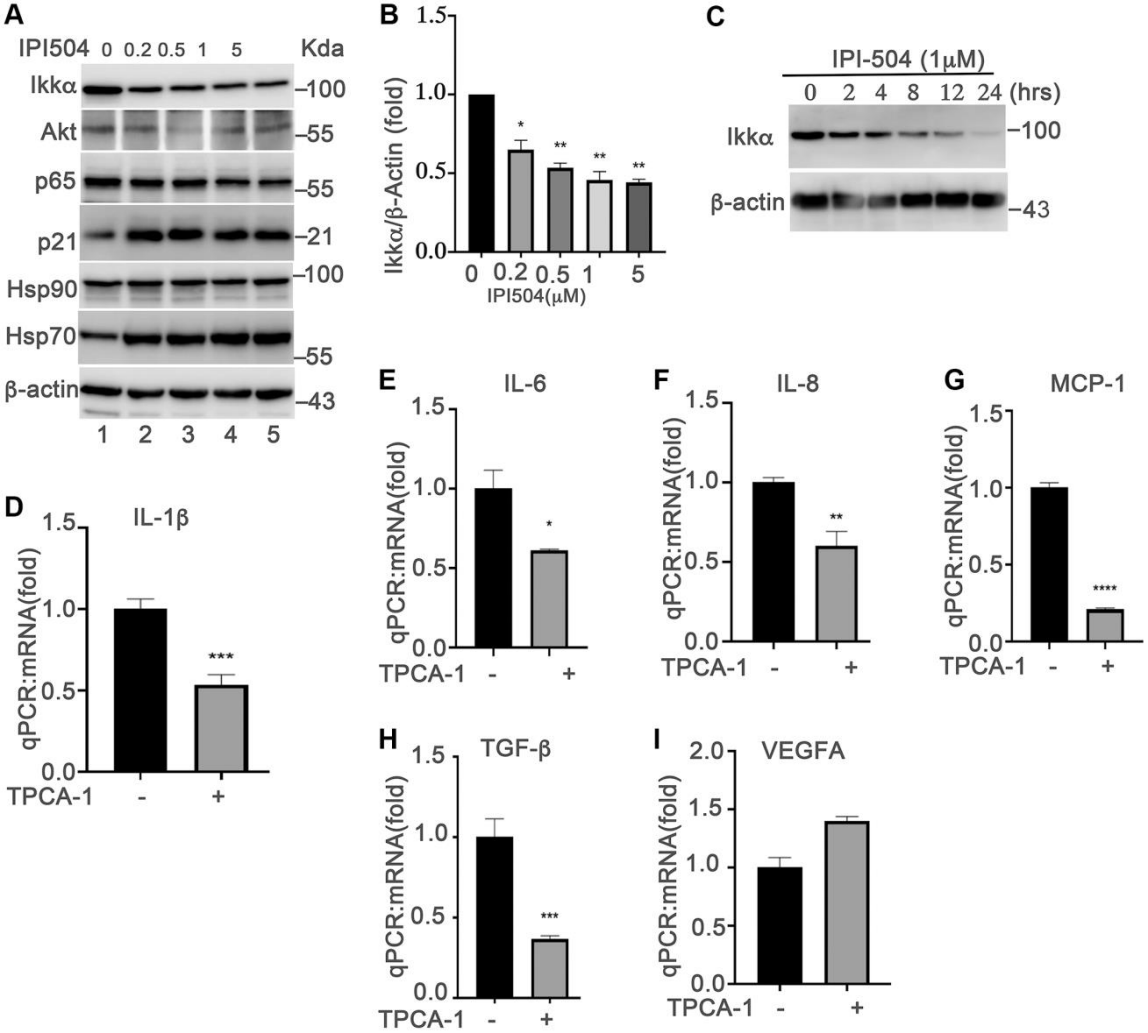
**NF-kB pathway is involved in HSP90-regulated expression of senescence-associated cytokines in senescent RPE cells *in vitro*.** (**A**) Immunoblot of IKKα AKT, p65, p21, HSP90, HSP70, HSC70 and β-actin in day-4 senescent ARPE-19 cells treated with PBS (sham) (lane 1) or IPI-504 at concentrations 0.25, 0.5, 1 and 5 μM (lanes 2–5). (**B**) Densitometry quantitation of IKKα in A. The results shown are mean ± SD. The two-tailed unpaired *t*-test was used for statistical analysis (*n* = 4). (**C**) Immunoblot of IKKα and GAPDH in day-4 senescent ARPE-19 cells that were treated with media containing PBS (sham, lane 1), 1 μM IPI-504 for 2, 4, 8, 12, 24 hours (lanes 2–6). (**D**–**I**) mRNA expression of IL-1β, IL-6, IL-8, MCP-1, TGF-b and VEGFA in day-4 senescent ARPE-19 cells treated with or without IKKα/IKKβ inhibitor TPCA-1. The data were collected from 5 independent experiments, the two-tailed unpaired *t*-test was used for statistical analysis, ^*^p < 0.05, ^***^p < 0.001.

Upregulation of SASPs is a fundamental mechanism of senescent cells that is responsible for aging and aging-associated diseases. NF-κB pathway is the predominant regulator of cytokines in senescent cells [19]. The senescence-inducers (e.g., ROS) activate the IKKα/ IKKβ complex to phosphorylate and induce the degradation of iκB, which results in the translocation of P65 into the nucleus to upregulate the expression of cytokines. Inhibition of HSP90 by geldanamycin induces IKKα and IKKβ degradation, and inhibits the constitutive and inducible NF-κB activity [37]. Thus, we postulated that HSP90 regulates the SASPs in senescent ARPE-19 cells by associating with NF-κB pathways. To test this hypothesis, we treated day-4 senescent ARPE-19 cells with IPI-504 at the indicated concentrations (Figure 4A). The results showed that IPI-504 reduced the protein levels of IKKα and AKT in a dose- and time-dependent manner (Figure 4A–4C, and Supplementary Figure 5A, lane 2), and this reduction of IKKα was restored by lysosomal inhibitor chloroquine but not by MG132 (Supplementary Figure 5A, lane 3), suggesting that IPI-504 induced IKKα protein degradation occurred in the lysosome, which is consistent with a previous report [38]. As a consequence, IPI-504 induced iκB protein expression (Supplementary Figure 5A, lane 2), and inhibited p65 nuclear translation (Supplementary Figure 5B). These results suggested that IPI-504 inhibited NF-κB pathway activities in senescent RPE cells. To confirm whether the NF-κB pathway regulates the SASP phenotype in senescent ARPE-19 cells, we treated day-4 senescent ARPE-19 cells with TPCA-1, an inhibitor of IKK1β and IKK1α. TPCA-1 inhibited the expression of IL-1β, IL-6, IL-8 and MCP-1, but not VEGFA in senescent ARPE-19 cells (Figure 4D–4I) as well as in replicative senescent monkey RPE cells (Supplementary Figure 5C). Taken together, these results indicated that the NF-κB pathway was activated for the expression of IL-1β, IL-6, IL-8 and MCP-1 during senescence in ARPE-19 cells, and the activities of the NF-κB pathway was regulated by HSP90 chaperone activity.

### HSP90 associates with and regulates SA-β-Gal activity in senescent RPE cells

**Figure 5:**
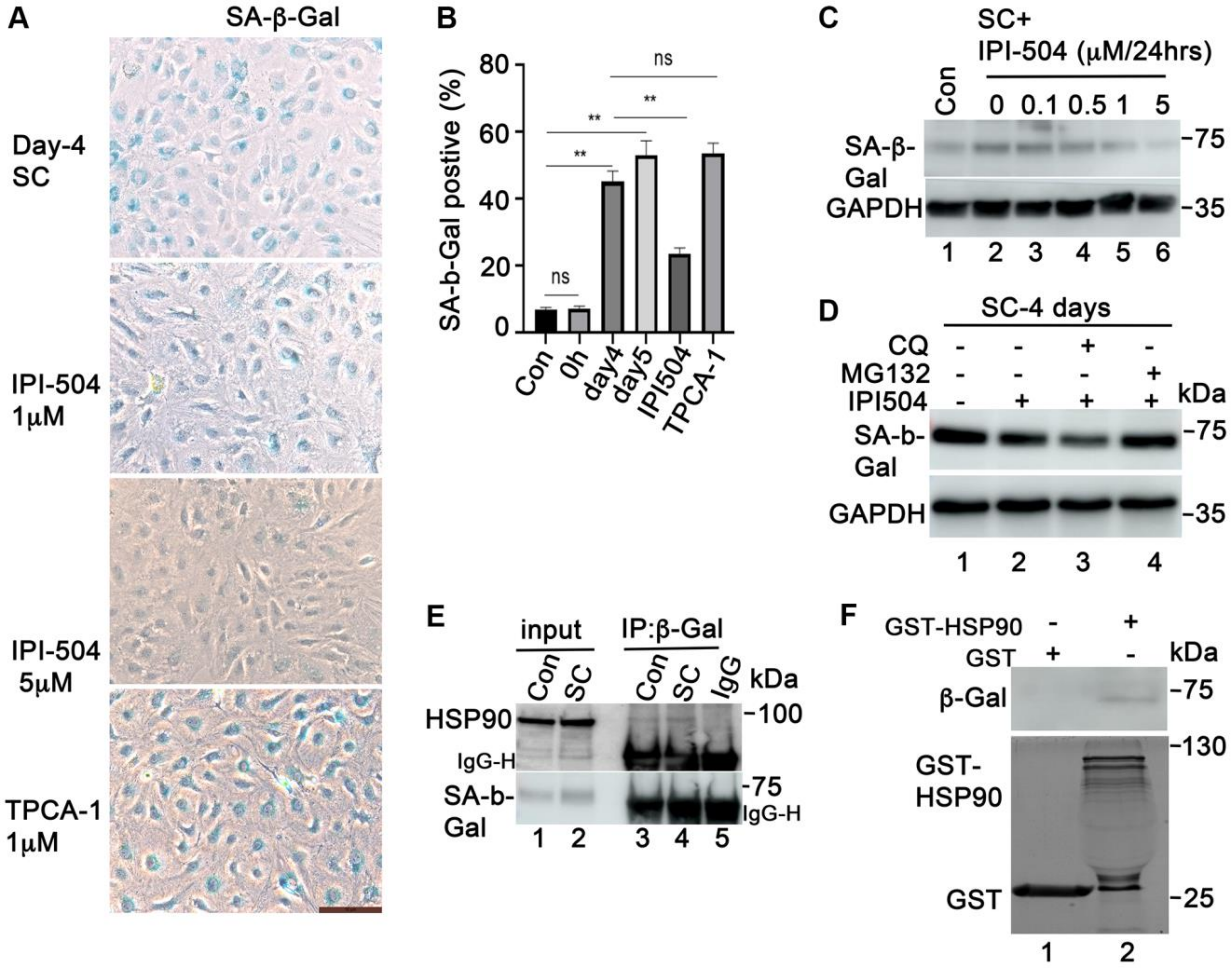
**HSP90 associates with and regulates SA-β-Gal in senescent ARPE-19 cells *in vitro*.** (**A**) SA-β-Gal staining the passage 3 primary monkey RPE cells treated with H_2_O_2_ for 2 h followed by recovery in normal media for 5 days. IPI-504 (1 and 5 μM) or TPCA-1 (1 μM) were added to day 4 recovery cells for 24 hours. (**B**) Percentage of SA-β-Gal positive cells out of total number of cells in A. Numbers were derived as an average from 5 different fields of view from three independent experiments. (**C**) Immunoblot of SA-μ-Gal protein in proliferating ARPE-19 cells (Con, lane 1) or the senescent ARPE-19 cells treated with IPI-504 at 0, 0.1, 0.5, 1 and 5 μM for 24 hours. (**D**) MG132 rescued the down-regulation of SA-β-Gal protein by IPI-504 in senescent ARPE-19 cells. (**E**) Immunoprecipitation assay to determine the interaction between HSP90 and SA-β-Gal protein in control (lane 3) and senescent ARPE-19 cells (lane 4). Lanes 1–2 are cell lysate controls, lane 5 is IgG control. (**F**) GST-pull down assay to determine the interaction between SA-β-Gal proteins and bacterially purified GST-HSP90 in senescent ARPE-19 cells. Upper panel shows the SA-β-Gal protein in day 4 senescent ARPE-19 cells that co-precipitated with bacterially expressed GST-HSP90α fusion protein (lane 2) or GST protein alone (lane 1). The lower panel is the coomassie blue stain of the bacterially purified GST and GST-HSP 90α.

Upregulation of the enzymatic activity of lysosomal SA-β-galactosidase is a dominant hallmark of senescent cells. To determine whether IPI-504 exerts any regulatory effect on SA-β-Gal activity, ARPE-19 cells were treated with 200 μM H_2_O_2_ for two hours followed by recovery in complete media for 1, 2, 3 and 4 days. After 24 hours recovery, 1 μM IPI-504 or PBS (sham) was added to the media for 24, 48, and 72 hours. The SA-β-Gal activity was stained. The results showed that in the sham group, most of the cells flattened and expanded in size, and was SA-β-Gal positive. However, IPI-504 treated cells shrank with long, protruding filipodia and exhibited less SA-β-Gal staining (Supplementary Figure 6A). The immunoblotting results indicated that caspase 3 was not activated (data not shown). These results suggested that IPI-504 reduced SA-β-Gal activity in senescent cells. Furthermore, we tested this potential inhibitory effect of IPI-504 on SA-β-Gal activity in H_2_O_2_-induced senescent primary monkey RPE cells. Primary monkey RPE cells at passage 3 were treated with H_2_O_2_ for 2 hours followed by recovery in normal media for up to 5 days. IPI-504 was added to day-4 senescent cells for 24 hours, and SA-β-Gal activity was measured (Figure 5A). The quantitation results showed that 53–57 % of cells on day 4 or 5 of recovery after H_2_O_2_ treatment were SA-β-Gal positive (Figure 5B), while only 58% SA-β-Gal positive cells observed in controls or in cells without recovery after H_2_O_2_-treatment. IPI-504 at 5 μM significantly reduced SA-β-Gal activity without impacting the total cell numbers (Figure 5A and 5B), whereas the IKKα/IKKβ inhibitor TPCA-1, which inhibited senescent SASP (Figure 4 and Supplementary Figure 5), did not affect SA-β-Gal activity (Figure 5A). Taken together, these results suggested that HSP90 chaperone activity is associated with SA-β-Gal activity in senescent primary RPE cells.

SA-β-Gal is a lysosomal enzyme that hydrolyzes β-galactosidase into monosaccharides in senescent cells and in some post mitotic cells [39]. Its activity is correlated with its expression level in senescent cells [40]. We further studied the expression of SA-β-Gal in the cells that were treated with IPI-504. The results showed that SA-β-Gal protein was upregulated in day-4 senescent ARPE-19 cells compared to that in normal ARPE-19 cells (Figure 5C, lanes 1 and 2). IPI-504 decreased SA-β-Gal protein levels in a dose-dependent manner in day-4 senescent ARPE-19 cells (Figure 5C, lanes 2–6) without affecting SA-β-Gal mRNA levels (Supplementary Figure 6B). The reduction of SA-β-Gal by IPI-504 was restored by proteasome inhibitor MG132, but not by lysosome inhibitor chloroquine (Figure 5D). The decrease in SA-β-Gal protein level was also observed in IPI-504-treated HeLa cells (Supplementary Figure 6C). These results indicated that HSP90 is involved in regulating SA-β-Gal protein stabilization. We further tested the protein-association between HSP90 and SA-β-Gal using GST-pull down and immunoprecipitation assays. HSP90 coimmunoprecipited with SA-β-Gal in both proliferating and senescent ARPE-19 cells (Figure 5E). The GST-pull down results showed that SA-β-Gal proteins in senescent ARPE-19 cells co-precipitated with bacterially purified GST-HSP90 proteins but not with GST protein alone (Figure 5F, lanes 2 and 1). The GST and GST-HSP90 proteins used for pull down assay were stained in coomassie blue (Figure 5F, low panel) Taken together, these results suggested that HSP90 interacted with and regulated β-galactosidase protein stability in senescent RPE cells.

### HSP90 upregulates VEGFA expression in senescent ARPE-19 cells by associating with HIF1α

**Figure 6:**
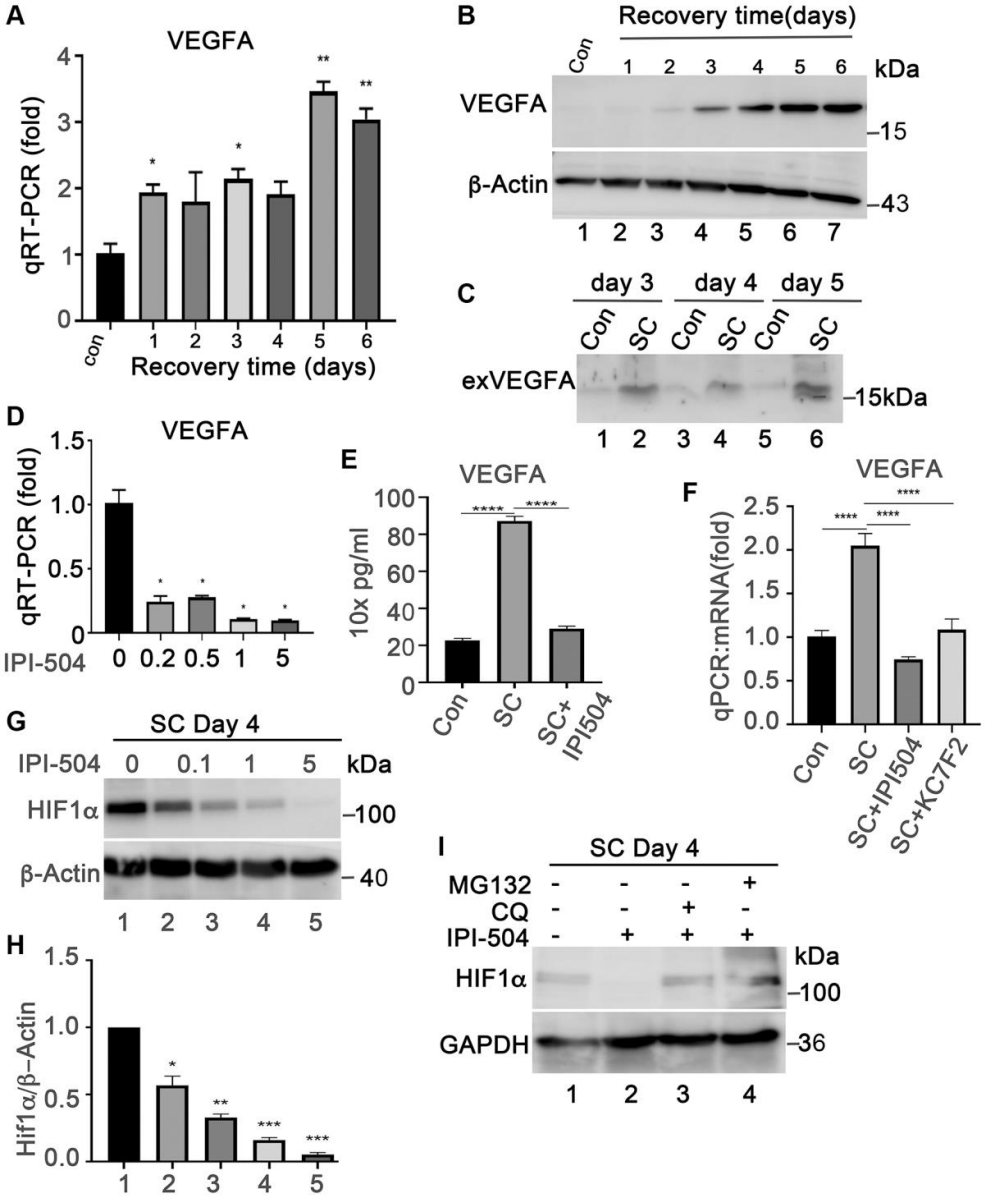
**IPI-504 inhibits VEGFA expression by down-regulating HIF1α protein expression in senescent RPE cell *in vitro*.** (**A**) Quantitative PCR determined VEGFA expression in control ARPE-19 cells (Con) or ARPE-19 cells treated with 2 h H_2_O_2_ followed by recovery in normal media for 0, 1, 2, 3, 4 and 5 days. (**B**) Immunoblot of VEGFA protein in the cells used in A. (**C**) Immunoblot of VEGFA in the supernatant of proliferative ARPE-19 cells (Con) or senescent ARPE-19 cells that were cultured for 3, 4 and 5 days. (**D**) Quantitative PCR determine VEGFA mRNA in day 4 senescent ARPE-19 cells treated with IPI-504 at 0.2, 0.5, 1 and 5 μM. (**E**) ELISA determines the VEGFA protein in the supernatants of day 4 senescent ARPE-19 cells treated with 1 μM IPI-504 for 24 hours. (**F**) Quantitative PCR determines VEGFA mRNA expression in day 4 senescent ARPE cells treated with HIF1α inhibitor KC7F2 or HSP90 inhibitor IPI-504. The data in each quantitation figure were collected from three independent experiments, the two-tailed unpaired *t*-test was used for statistical analysis, ^*^P < 0.05, ^***^p < 0.001. (**G**) Immunoblot of HIF10α protein in day-4 senescent ARPE-19 cells treated with IPI-504 at 0, 0.1, 1 and 5 μM. β-actin was used for protein loading control. (**H**) Densitometry quantitation of HIF1α vs. β-actin in G, the data shown are mean ± SD. The two-tail unpaired *t*-test was used for statistical analysis (*n* = 3). ^*^P < 0.05; ^***^P < 0.001. (**I**) Immunoblot HIF1α and GAPDH proteins in senescent ARPE-19 cells treated in media containing PBS (sham, lane 1), IPI-504 alone (lane 2), IPI-504 +chloroquine (lane 3) and IPI-504 + MG132 (lane 4).

Senescent RPE is a source of VEGFA, a key angiogenetic factor driving the progression of wet AMD. We measured the expression VEGFA in ARPE-19 cells that were treated with H_2_O_2_ in the same way as in Figure 1B using both qPCR and immunoblotting. The results showed that VEGFA mRNA was induced in H_2_O_2_-treated ARPE-19 cells as compared to normal ARPE-19 cells (Figure 6A). Consequently, the results of immunoblots and ELISA indicated that the expression of VEGFA protein was induced and secreted into the supernatants during cells undergoing senescence (Figure 6B, 6C and 6E). These results indicated that senescent ARPE-19 could produce more VEGFA than proliferating ARPE-19 cells, which is consistent with previous reports [41]. To determine whether IPI-504 suppresses VEGFA expression in senescent RPE cells, day-4 senescent ARPE-19 cells were treated with IPI-504 at 0, 0.2, 0.5, 1 and 5 μM for 24 hours. The qPCR and ELISA results indicated that IPI-504 efficiently inhibited the expression of VEGFA at both mRNA (Figure 6D and 6F) and protein levels (Figure 6E). In contrast, inhibition of IKKα/IKKβ with TPCA-1 slightly increased VEGFA mRNA expression without statistical significance (Figure 4I). These results suggested that IPI-504 inhibited the expression and secretion of VEGFA in senescent ARPE-19 cells and this inhibition is not mediated by the NF-κB pathway.

HIF1α is a transcription factor that regulates VEGFA transcription under hypoxia or oxidative stress conditions. HIF1α is a client protein of HSP90. Thus, we postulated that the inhibition of VEGFA expression by IPI-504 was mediated through HIF1α. As expected, IPI-504 did not impact HIF1α mRNA expression (Supplementary Figure 7A), but it inhibited HIF1α protein expression in a dose dependent manner in day-4 senescent ARPE-19 cells (Figure 6G and 6H). The reduction of HIF1α by IPI-504 was restored by both lysosomal inhibitor chloroquine and proteasomal inhibitor MG132 (Figure 6I). These results suggested that HSP90 modulated HIF1α protein stability in senescent RPE cells. To determine whether HIF1α is responsible for VEGFA expression, we treated senescent RPE cells with the HIF1α inhibitor KC7F2, a chemical that inhibits HIF1α protein synthesis [42]. Like IPI-504, KC7F2, which inhibits protein synthesis of HIF1α (Supplementary Figure 7B), significantly inhibited VEGFA mRNA expression in day 4 senescent ARPE-19 cells (Figure 6F). These results suggested that IPI-504 inhibited VEGFA expression in senescent ARPE-19 cells by decreasing HIF1α protein stability.

### IPI-504 attenuates senescent ARPE-19-mediated cell migration

**Figure 7:**
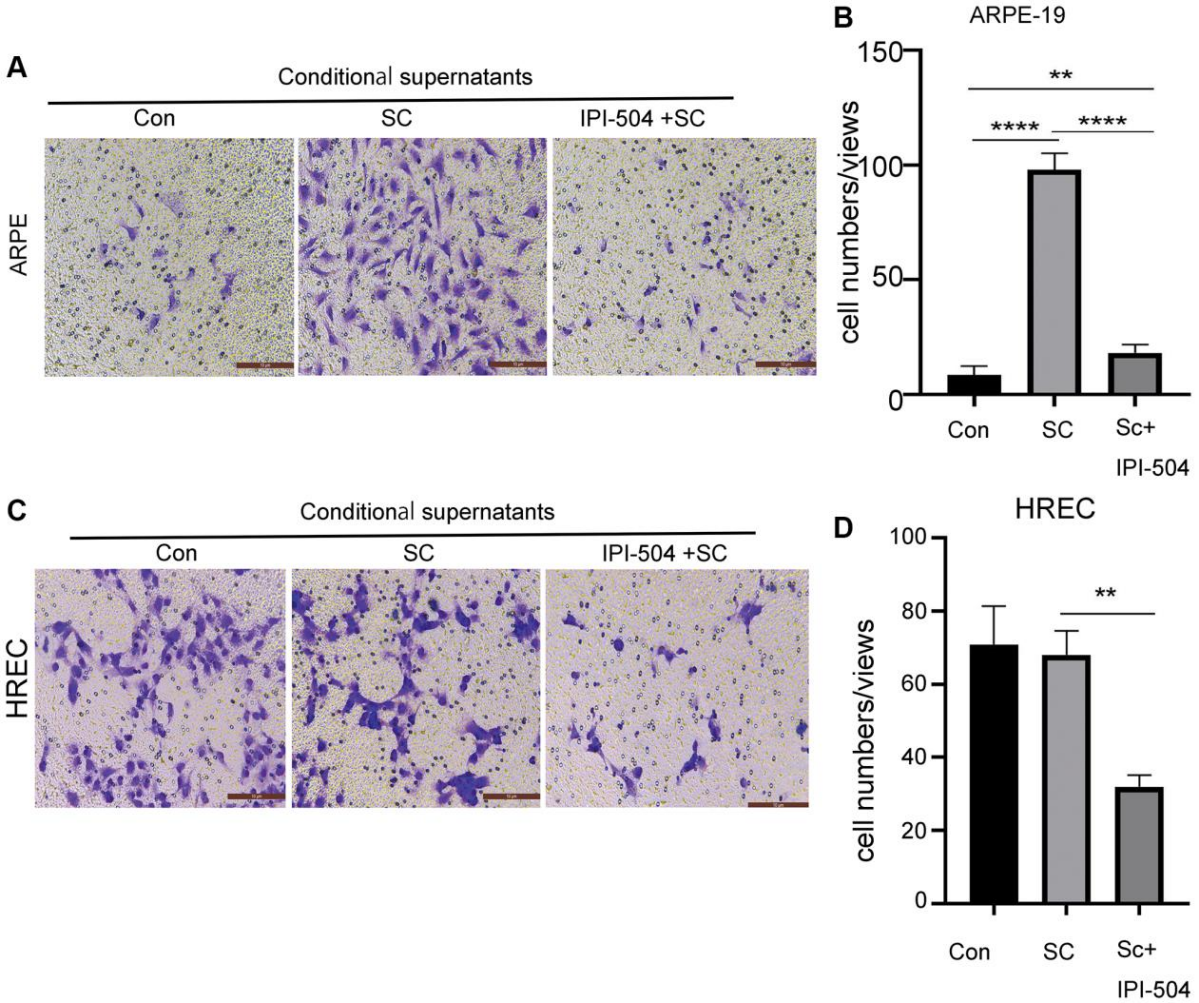
**IPI-504 inhibit senescent ARPE-19 cells-mediated cell migration.** (**A**) Transwell assay. The chamber with ARPE-19 cells were incubated in wells containing the conditional media from proliferating ARPE-19 cells, day-4 senescent ARPE-19 cells and day 4-senescent ARPE-19 cells that were pretreated with IPI-504 for 24 hours. The migrated cells at bottom were photographed. (**B**) The quantitation of migrated cell numbers in A. (**C**) Transwell assay. The chamber with HREC cells were incubated in wells that contains the conditional media used in A. The migrated cells at bottom were photographed. (**D**) The quantitation of migrated cell numbers in C. The two-tailed unpaired *t*-test was used for statistical analysis (*n* = 4).

Senescent RPE cells associate with its neighbor or distant cells via SASP. One of SASP function is to regulate cell migration. Because IPI-504 inhibits the SASP in senescent RPE cells, we postulated that IPI-504 suppressed senescent ARPE-mediated cell migration. To this end, day-4 senescent ARPE-19 cells were treated with media containing IPI504 or PBS (sham) for 24 hours respectively. After this, the cells were washed with PBS and continually cultured in serum-free media for 24 hours. The supernatants were collected as conditional supernatants to incubate ARPE-19 cells and HREC (human retinal endothelial cells) cells for migration test. The results indicated that the supernatants of senescent ARPE-19 cells increased migration of ARPE-19 but not HREC cells compared to the supernatants from normal ARPE-19 culture (Figure 7). The supernatants of IPI-504-pretreated senescent ARPE-19 cells significantly attenuated the migration of ARPE cells and HREC cells compared to the supernatants of senescent ARPE-19 cells (Figure 7A–7D, and Supplementary Figure 8). Taken together, these results suggested that IPI-504 could attenuate the senescent ARPE-19 cell’s communication with its neighboring or distant cells.

## DISCUSSION

The senescent RPE cells have been considered a new target for AMD intervention or prevention [7]. There are two types of senotherapies, senolytics, which induce senescent cells to undergo apoptosis, and senomorphics, which reduce levels of senescence-associated inflammatory factors in senescent cells to decrease chronic inflammation [22]. Both options are able to effectively improve aging and aging-associated diseases (e.g., cancer, arthritis and atherosclerosis, fibrosis) in animal models. In this paper, we studied the senotherapeutic role of HSP90 in senescent ARPE-19 cells and primary monkey RPE cells *in vitro*. We found that: 1) The expression of heat shock proteins is reprogrammed during senescence (Figure 2); 2) Senescent ARPE-19 cells are more resistant to the cytotoxic effects of the HSP90 inhibitor IPI-504 when compared to the proliferating ARPE-19 cells (Supplementary Figure 3). Administration of IPI-504 at low doses significantly reduced the expression and secretion of senescence associated inflammatory factors including IL-1β, IL-6, IL-8, MCP-1 and VEGFA, and SA-β-Gal activity in senescent RPE cells. As consequence, IPI-504 attenuated the senescent ARPE-19 cell-mediated endothelial cell migration. These results suggest that HSP90 is a promising candidate as a senomorphic target for AMD intervention.

Accordingly, HSP90 exhibits divergent roles in regulating aging and age-associated diseases depending on the cell type and state. On one hand, deletion of HSP90 beta, but not HSP90 alpha, is embryonic lethal and promotes muscle cell senescence in mice by modulating the MDM2-P53-P21 pathway [30]. HSP90 beta deletion also increases a cell’s sensitivity to oxidative stresses. Furthermore, inhibition of HSP90 in p14-upregulated cancer cells leads to cell apoptosis and increases the cell’s sensitivity to DNA-damage [43]. On the other hand, HSP90 is also involved in regulating the aging process. Inhibition of HSP90 prolongs the lifespan of Errc-1 deficient progeroid mice [28] by inducing the apoptosis of senescent cells. In addition, inhibition of HSP90 reduces the injury of amyloid-fibrillin to neurons, and improves memory in Alzheimer disease [44]. In the retina, HSP90 is induced in retinitis pigmentosa (RP), the most common form of inherited photoreceptor degeneration caused by a mutation in the rhodopsin gene [23]. HSP90 inhibitor 17-N-allylamino-17-demethoxygeldanamycin (17-AAG) can protect against rhodopsin aggregation and toxicity in a cell model of a class II misfolding mutation in rhodopsin, P23H, and improve visual function and photoreceptor survival for several weeks in a P23H-rhodopsin transgenic rat model. One possible mechanism is through activation of the HSF1-mediated heat shock response [45]. In *C. elegans*, inhibition of HSP90 extends lifespan [27]. According to our data, we postulated that HSP90 is an undispersed chaperon molecule in senescent RPE. Although HSP90 expression did not change between normal proliferative and senescent RPE, the inhibition of HSP90 by its specific inhibitor IPI-504 at less cytotoxic dose, effectively inhibited the mRNA expression of senescence associated inflammatory factors such as IL-1β, IL-6, IL-8, MCP-1 and VEGFA (Figure 3, Supplementary Figure 4 and Figure 6) without inducing cell apoptosis (data not shown). These results suggest that HSP90 acts as a senomorphic target in senescent RPE. Our data show that HSP90 regulates the SASP in senescent RPE through at least two different signaling pathways, NK-κB and HIF1α (Figure 4 and Figure 6). The data in Figure 4 indicated that IPI-504 reduces IKKa and AKT protein expression (Figure 4A–4C) [38] which subsequently inhibits P65 activation (Supplementary Figure 5). This leads to the downregulation of IL-1β, IL-6, IL-8 and MCP-1, but not VEGFA (Figure 3 and Supplementary Figure 4). These results suggest the NF-κB pathway is involved in HSP90 mediated cytokine production (i.e., IL-1b, IL-6, IL-8 and MCP-1) but not VEGFA. It was reported that inhibition of HSP90 could inhibit the hypoxia-induced expression of VEGFA in RPE cells *in vitro* [34]. The hypoxia-inducible factor-1alpha (HIF1α) is a key transcription factor that senses hypoxic conditions and controls the expression of VEGFA. HSP90 is involved in regulating HIF1α protein stabilization by competing with the RACK1-elongin pathway [46]. The data in Figure 6 indicate that HSP90 is essential for VEGFA expression and secretion in senescent RPE cells. The inhibition of HSP90 or HIF1α reduces VEGFA’s mRNA expression and protein secretion respectively, and inhibition of HSP90 reduces HIF1α protein expression without affecting its mRNA levels (Figure 6). These data suggest that HSP90 regulates VEGFA expression by modulating HIF1α protein stability in senescent RPE cells.

Senescent cells communicate with its neighboring or distant cells via SASP including cell’s migration. The results in Figure 7 indicate that IPI-504 could attenuate senescent ARPE-19 cells-mediated cell migrations of normal ARPE-19 and HREC endothelial cells *in vitro*. These results suggested that IPI-504 could be a promising candidate for AMD intervention.

Senescence-associated β-galactosidase (SA-β-Gal) is a hallmark of senescence although its biological functions are not fully elucidated. β-galactosidase is constitutively expressed in the lysosome and induced under senescence promoting conditions. Some studies suggest that SA-β-Gal is the consequence rather than the initiating factor in senescent cells [40]. Our data demonstrate that HSP90 is involved in regulating the SA-β-Gal activity in senescent RPE cells (Figure 5 and Supplementary Figure 6). SA-β-Gal protein is upregulated in senescent ARPE-19 cells (Figure 5D, lanes 1 and 2), and IPI-504 reduces SA-β-Gal protein expression without affecting its mRNA levels (Figure 5C, 5D, and Supplementary Figure 6B). The inhibition of β-galactosidase protein expression by IPI-504 is also observed in HeLa cells (Supplementary Figure 6C). The reduction of SA-β-Gal proteins by IPI-504 was inhibited by proteasome inhibitor MG132, but not by lysosomal inhibitor chloroquine (Figure 5D). These results suggested that HSP90 participated in regulating SA-β-Gal protein stabilization. Moreover, we show that HSP90 interacts with SA-β-Gal in both GST-pull down and immunoprecipitation assays (Figure 5E and 5F). Accordingly, we propose that HSP90 is involved in regulating SA-β-Gal protein stability in senescence, and SA-β-Gal is a novel client protein of HSP90. HSP90 has been shown to localize in the lysosome together with HSP73 in kidney proximal tubular epithelial cells [47]. Furthermore, inhibition of HSP90 has been shown to disturb endosome sorting and chaperone-mediated autophagy [48], which suggests that HSP90 mediates lysosomal activity. However, the biological significance of HSP90’s regulation on SA-β-Gal protein expression is still under investigation.

## CONCLUSIONS

RPE undergo senescence under stressful conditions. Accumulation of senescent RPE in the retina has been linked to diseases such as acute macular degeneration. HSP90 maintains homeostasis of senescent RPE in part by regulating the SASPs and SA-β-Gal activity. HSP90 is a promising target for senomorphic intervention in senescent RPE cells.

## MATERIALS AND METHODS

### Chemical reagents and antibodies

The IPI-504 was purchased from ApexBio Technology (Houston, TX, USA). TPCA-1 and MG132 were from Med-Chem Express (Monmouth Junction, NJ, USA), KC7F2 was from Selleck Chemicals (Houston, TX, USA). The rabbit polyclonal antibodies against HSP27 (HSPB1) and ß-actin were purchased from Sigma-Aldrich (St. Louis, MO, USA). The rabbit antibody against αB-crystallin was from Santa Cruz Biotechnology (Dallas, TX, USA). The antibodies against HSP90, HSP70, HSP27, p21, p53, IKKα, and AKT were purchased from Cell Signalling Technology (Shanghai, China). The rabbit antibodies for iκB, GAPDH and p65 were from Proteintech (Wuhan, China). The senescence associated β-galactosidase staining kit was bought from Beyotime Institute of Biotech (Shanghai, China).

### Cell lines

ARPE-19 cells were ordered from ATCC (Manassas, VI, USA). The rhesus monkey primary RPE cells were isolated from retinas of 2 years old female rhesus monkeys, which were used for acute myocardial infraction experiments by another lab [49]. The protocols for harvesting PRE cells from the rhesus monkeys were approved by the Zhengzhou University ethics committee. The ARPE -19 and primary RPE cells were cultured in DMEF/F12 media containing 10% FBS with 1x ampicillin and streptomycin.

### Isolation of primary monkey RPE cells

The eye balls were enucleated from 2 years old female rhesus monkeys that were used for an acute myocardial infarction assay [49]. The cornea and lens were removed from the eye balls, and contents from the posterior eye cup including the retina, pigment epithelium, choroid and sclerosis were collected and rinsed in PBS and DMEM/F12 media. The retina was separated from the choroid and sclera, and the remainder of the eye cup was cut and flatted. The RPE tissue was scraped, and the cells were suspended in complete media containing 10% FBS and antibiotics. 10^6^ cells were seeded into each 10 cm culture dish. The cells were passaged when they reached 80% confluence.

To achieve replicative senescence, the primary RPE cells were passaged for 8–10 times until the cells lost proliferation potential.

### The induction of senescent RPE cells by H_2_O_2_ *in vitro*

The ARPE-19 cells or monkey primary RPE cells at passage 3 were cultured in complete media overnight. H_2_O_2_ was added to cells at a concentration of 200–300 μM for 2 hours. The media was subsequently replaced with fresh complete media, and the cells were cultured continuously up to 10 days with fresh media change every other day. Senescence markers were measured in these cells. For the IPI-504 treatment, the senescent RPE cells at day 4 or day 5 post H_2_O_2_-treatment were incubated with media containing 0.1–5 μM of IPI-504 for 24 hours.

### SA-ß-Gal staining assay

RPE cells were seeded into 12-well plates and incubated overnight. Cells were treated with sham or H_2_O_2_ for 2 h, followed by recovery in complete medium for the indicated time. The cells were fixed in buffer containing 0.5% glutaraldehyde and 2% paraformaldehyde in PBS for 20 min. After this, the cells were incubated with a solution containing X-gal for 24 h at 37°C to measure SA-ß-Gal activity. SA-ß-Gal positive signals were photographed under the microscope (Zeiss, Oberkochen, Germany). The percentage of SA-ß-Gal positive cells were calculated by dividing the number of SA-ß-Gal positive cells by the total cell number in five different views. The data collected from three independent experiments were used for statistical analysis by using an unpaired two-tailed *t*-test.

### Quantitative real-time PCR (qRT-PCR)

Total RNA was extracted with RNAiso reagent following the manufacturer’s protocol (Takara, Beijing, China). One microgram of total RNA was used to synthesize cDNA (Takara). Equal amounts of cDNA were mixed with Faststart Universal SYBR Green Master Mix (Roche, San Francisco, CA, USA). qQRT-PCR was performed using an ABI 7500 system (Applied Biosystems, Foster City, CA, USA). The primers for detecting mouse p21^cip1^, p53^ink4^, and senescence-associated inflammation factors (IL-1, IL-6, IL-8, MCP-1, TGF-b, VEGFA, et al.) are listed in Table 1. The data shown were mean ± SD (*n* = 4). The unpaired two-tailed *t*-test were used for statistical analysis. A *p* < 0.05 was considered statistical significance.

**Table 1:** The primers used for quantitative PCR.

**Primer name**	**Forward primer (5′ to 3′)**	**Reverse primer (5′ to 3′)**
IL-1β	AGTACCTGAGCTCGCCAGT	TGGTGGTCGGAGATTCGTAG
IL-6	TGAACTCCTTCTCCACAAGCG	CCGTCGAGGATGTACCGAAT
IL-8	GCTCTGTGTGAAGGTGCAGTT	ACCCAGTTTTCCTTGGGGTC
MCP	CGCCTCCAGCATGAAAGTCT	AGGTGACTGGGGCATTGATT
P21	AAGTCAGTTCCTTGTGGAGC	GCCATTAGCGCATCACAGTC
P53	ACCTATGGAAACTACTTCCTGAAA	CTGGCATTCTGGGAGCTTCA
BCL-XL	CCTAAGGCGGATTTGAATAATCTT	CCAAAACACCTGCTCACTCAC
HSPB5	CTGAGTCCCTTCTACCTTCGG	ATCCTGGCGCTCTTCATGTT
HSP27	CACGCAGTCCAACGAGATCA	TTACTTGGCGGCAGTCTCAT
HSC70	TATTGGAGCCAGGCCTACAC	TCAGTGTCCGTAAAGGCGAC
HSP90α	GCTCCAAGGGTTGACATGGT	TGTAACTCATGGACGCAGGG
BIP	GAACGTCTGATTGGCGATGC	ACCACCTTGAACGGCAAGAA
VEGFA	CCCACTGAGGAGTCCAACATC	CTGCATTCACATTTGTTGTGCTG
C-FLIP	TGGTTCCACCTAATGTCA	GAGCAGTTCAGCCAAGTC
ACTB	ACCGCGAGAAGATGACCCAG	GGATAGCACAGCCTGGATAGCAA
GAPDH	GACAGTCAGCCGCATCTTCT	GCGCCCAATACGACCAAATC
HIF1α	TCAAAGTCGGACAGCCTCAC	GATTGCCCCAGCAGTCTACA
GLB1	TTCGCATCCTCCCTCTGTTG	TCAAACATCCTCTGGGTGGC

### ELISA assay

The senescent ARPE cells were treated with media containing PBS or IPI504 for indicated time. After this, the cells were then cultured in serum-free DMED-F12 for 24 hours. The supernatants were collected and applied to ELISA following kit’s provided protocol (Boster Bio, Pleasanton, CA, USA). The experiments were repeated independently for three times for statistical analysis of data (two-tailed unpaired *t*-test).

### Immunoblotting, immunoprecipitation, GST-pull down and immunofluorescent staining

The immunoblotting, immunoprecipitation, and GST-pull down assays have been described previously [50]. For immunoblotting, 30–50 μg of lysates were separated by sodium dodecyl sulfate polyacrylamide gel electrophoresis, and the proteins were transferred to polyvinylidene difluoride membranes. After 1 h of blocking in 5% skim milk/phosphate-buffered saline (PBS)/0.1% Tween 20, the membranes were incubated with primary antibodies at 4°C overnight. The membranes were washed with PBS/0.1% Tween-20 four times, then incubated with secondary antibodies conjugated with horseradish peroxidase for 1 h. The membranes were developed using enhanced chemiluminescence and exposed to X-ray film to detect signals.

For immunoprecipitation, 1 mg protein was precleared with protein A-agarose beads, followed by incubation with 2 μg of the primary antibody overnight. Protein A-agarose beads (50 μL) were added to the solution and incubated for an additional 2 h. The beads were washed 3–4 times with NP-40 lysis buffer. The co-precipitated products were used for immunoblotting.

For GST-pull down assay, 1 mg of cell lysis protein was incubated with 1 μg of bacterially purified GST or GTS-HSP90 proteins respectively overnight. The GST and GST-HSP90 proteins were precipitated with Glutathione Sepharose 4b beads. The coprecipitated products were applied to immunoblot with anti-β-galactosidase antibody. The bacterially purified GST and GST-HSP90 were detected by coomassie blue stain.

For immunofluorescent staining, The RPE cells were fixed in 3.7% Polyfolmaldehyde/PBS for 20 minutes, followed by permeabilization in 0.5% triton ×-100 for 2 minutes. The cells were then incubated with 2% BSA/PBS block buffer for 1 h followed by incubation with primary antibodies for 1 h. The cells were washed in PBST buffer 3 times and then incubated in buffer containing secondary antibody conjugated with HRP for 1 h. After mounting in buffer containing DAPI for nucleus staining, the fluorescent signals were photographed with a confocal microscope (Arial, Japan).

### Cell migration assays

For wound healing assay, ARPE-19 or human retina endothelial cells (HREC) were grown to a monolayer in 24 well plates. The cells were scratched with a sterile 10 ul-tip. The media were replaced with supernatants of proliferating ARPE-19, Day-5 senescent ARPE-19 or senescent ARPE-19 cells pretreated by IPI-504 for 24 hours. The scratched area was photographed at different time points and recovery area was calculated with image-J software. The percentage of wound closure was calculated. For transwell migration assay, the 3 × 10^4^ cells were seeded on the top layer membrane of a trans-well insert. The supernatants of control ARPE-19, senescent ARPE-19 cells or IPI-504-preteated ARPE-19 cells were collected as condition media and added to below the cell permeable membrane respectively. After 6–24 hours incubation, the inserts were washed once in PBS and fixed in 4% polyformaldehyde/PBS or 30 minutes following by fixing in 20% methanol for 10 minutes. The cells were stained in 10% crystal violet at room temperature for 10 minutes. After washing in H_2_O_2_ carefully, the inserts were air dried, and the top layer of cells were removed with cotton swab. The permeable cell number were accounted under microscope. The cell numbers were accounted in each views for total five different views. The data shown are mean ± SD (*n* = 5). The Unpaired two-tailed *t*-test was used for statistical analysis. A *p* < 0.05 was considered to be statistically significant.

### Statistical analysis

Image J was used to quantify the densitometry of immunoblot bands. SPSS 17.0 and GraphPad Prism 5 were used for data analysis. The two-tailed unpaired *t*-test was used for statistical analysis. *p* < 0.05 was considered to be statistically significant.

### Data available

All data generated or analyzed during this study are included in this manuscript and supplementary information files.

## Supplementary Materials

Supplementary Figures
