# Supplementary material for: HSP90 acts as a senomorphic target in senescent retinal pigmental epithelial cells
Source: Aging (Albany NY). 2021 Sep 8;13(17):21547–70. doi: 10.18632/aging.203496 (PMC8457597; doi:10.18632/aging.203496)
Supplement: Supplementary Figures [file aging-13-203496-s001.pdf]

SUPPLEMENTARY FIGURES

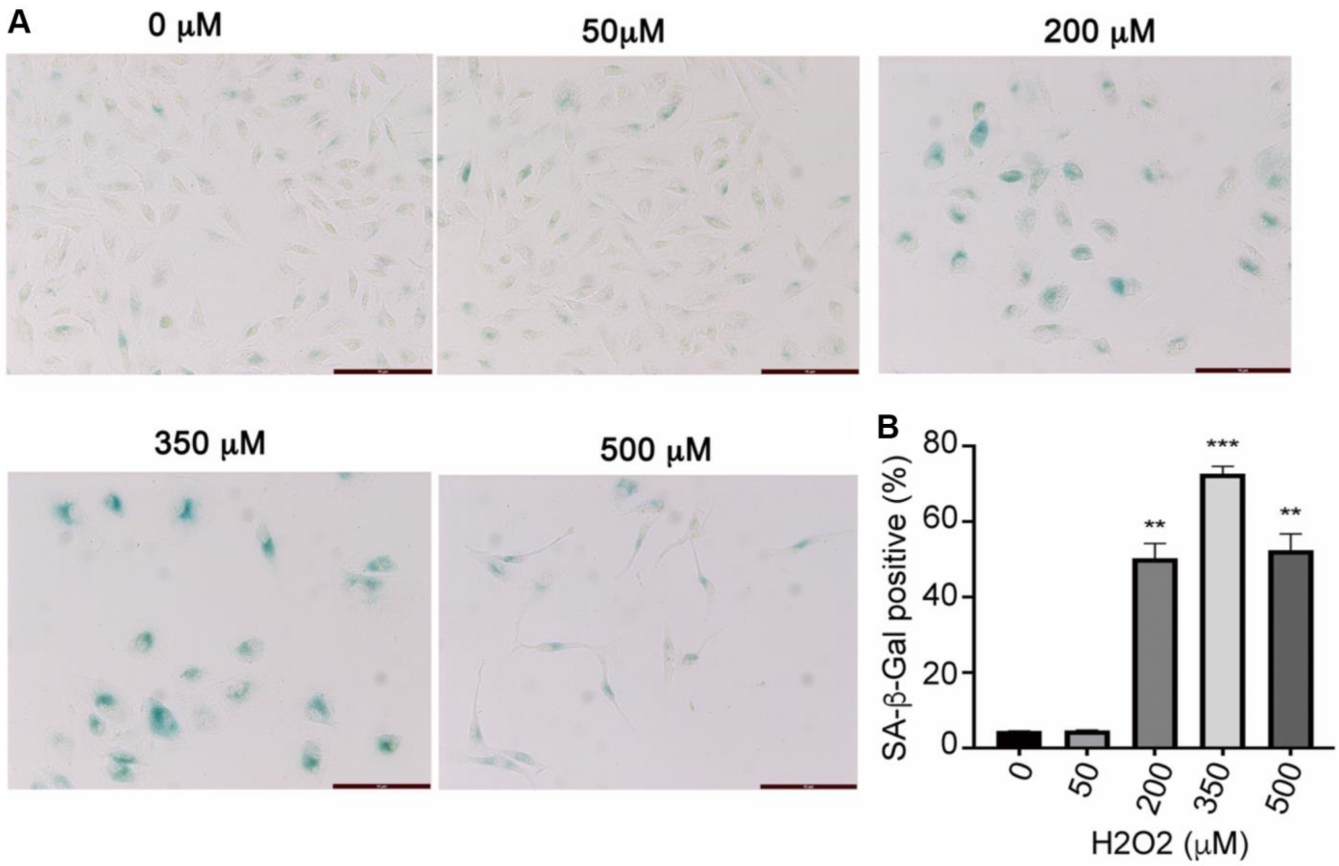

Supplementary Figure 1. H2O2 induces ARPE-19 cell senescence at different concentration. (**A**) The ARPE-19 cells were treated with H2O2 at 50, 200, 350 and 500 μM for 2 hours followed by recovery in normal media for 3 days. The activity of SA-b-Gal were stained. (**B**) The quantitation of SA-b-Gal positive cells vs. total cell numbers was counted for from five fields of a view in A. The data shown in bar graph are mean ± SD. The unpaired 2-tailed *t*-test was used for statistical analysis. *P* < 0.05 was considered to be statistical significant.

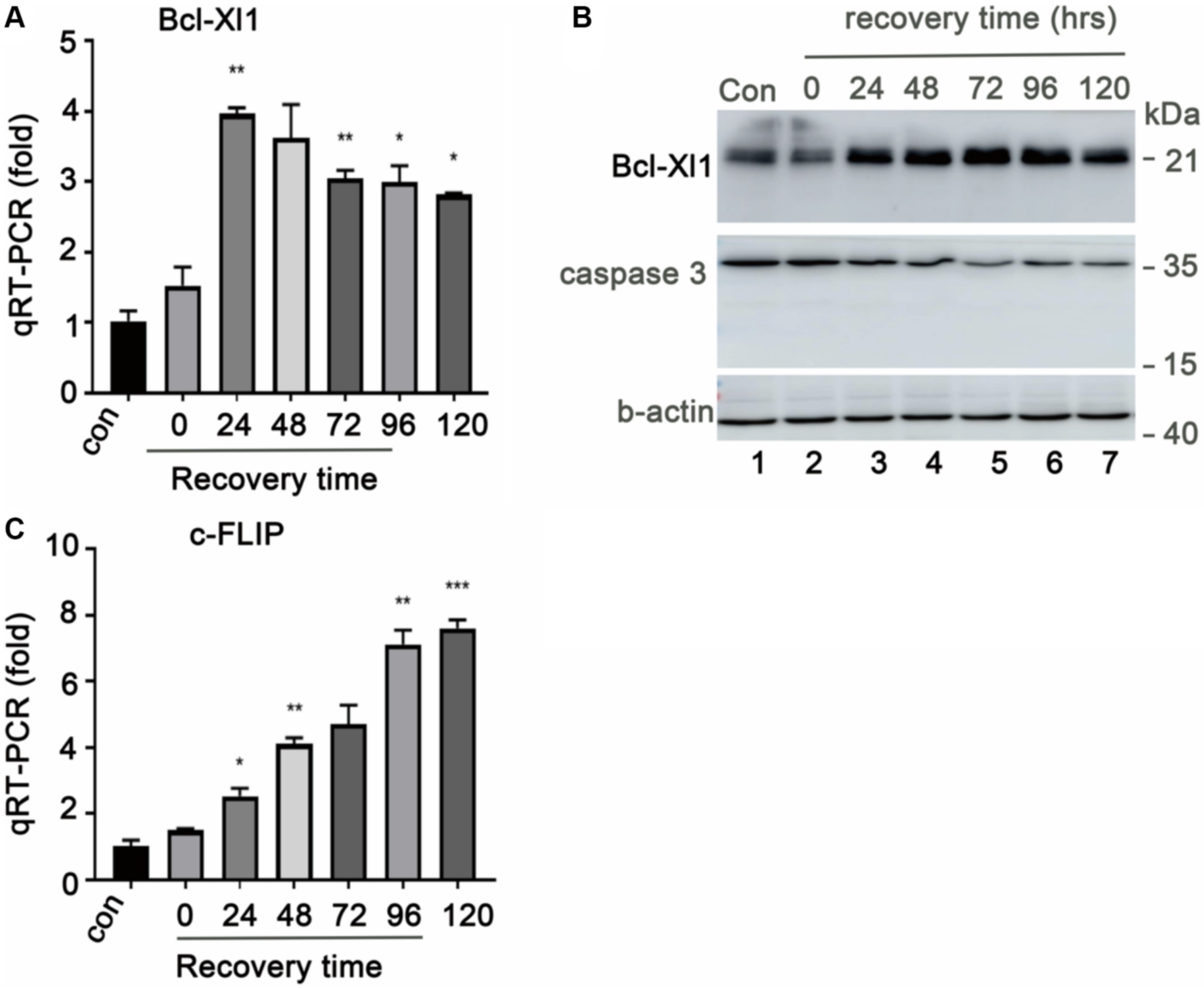

Supplementary Figure 2. The upregulation of anti-apoptotic proteins Bcl-xL and c-FLIP in senescent ARPE-19 cells. (**A**) Quantitative PCR to determine the expression of Bcl-xL mRNA in ARPE-19 cells at control condition (Con) or the cells treated with 200 µM H_2O_2 for 2 hours followed by recovery in normal media for 0, 24, 48, 72, 96 and 120 hours. (**B**) Immunoblot of Bcl-xL, Caspase 3 and b-actin proteins in the ARPE-19 cells treated in the same way as in A. (**C**) Quantitative PCR to determine the expression of c-FLIP in ARPE-19 cells treated in the same way as in A.

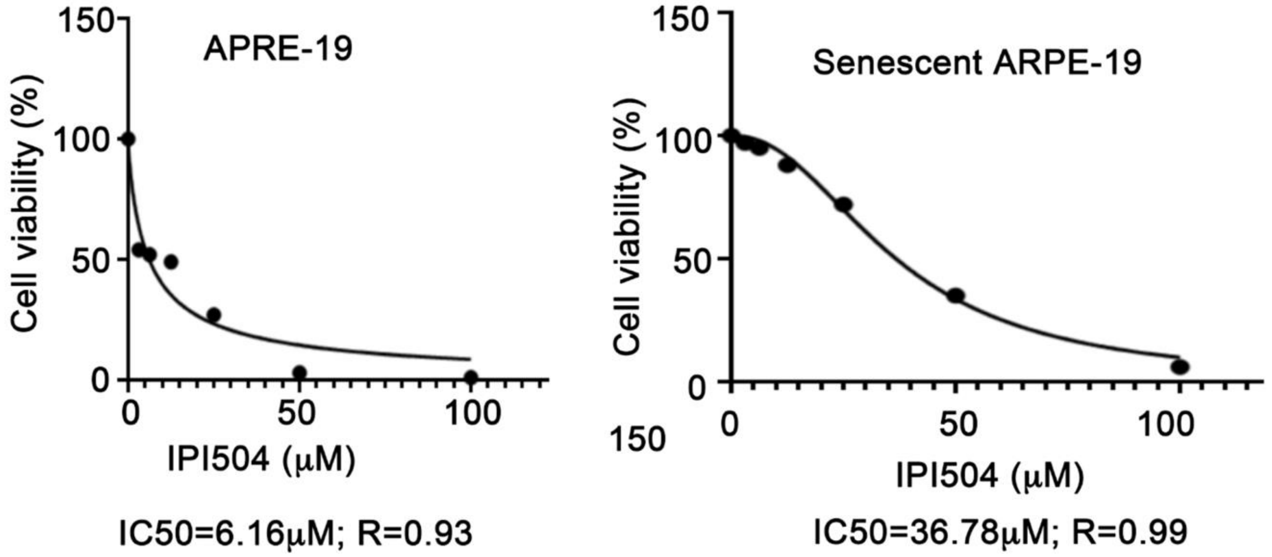

Supplementary Figure 3. The cytotoxicity of HSP90 inhibitor IPI-504 to the proliferating and senescent ARPE-19 cells. The proliferating and day-4 senescent ARPE-19 cells were treated with IPI-504 in different concentrations for 48 hours. The cell viability was measured with CCK-8 kit.

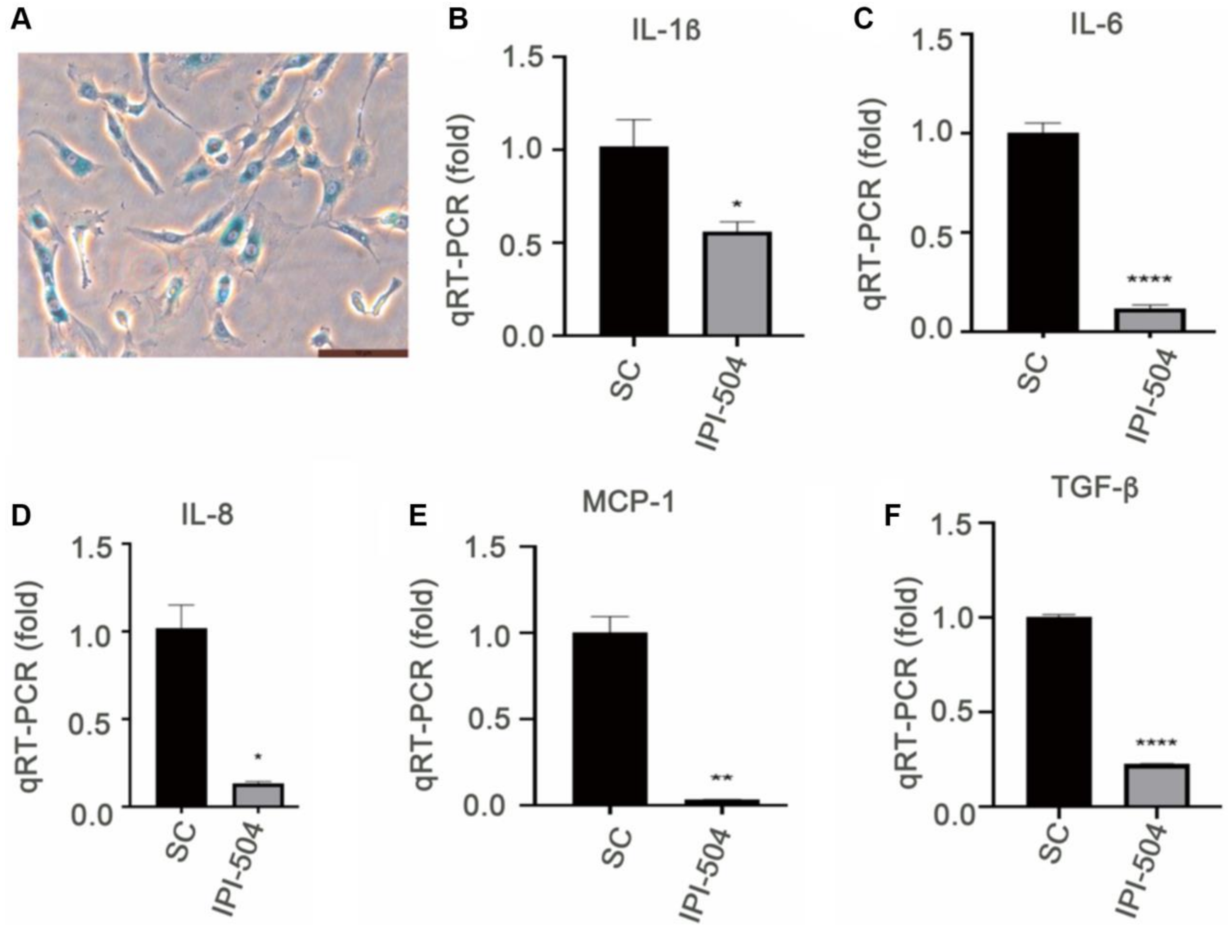

Supplementary Figure 4. IPI-504 inhibits mRNA expression of senescence-associated inflammatory factors in replicative senescent primary monkey RPE cells. (**A**) SA-b-Gal staining assay. The primary monkey RPE cells were cultured and passaged in DMEM/F12 media with 10% FBS for 8 generations. SA-b-Gal positive cells were stained. (**B**–**F**) Quantitative PCR to determine the mRNA expression of IL-1b, IL-6, IL-8, MCP-1 and TGF-b1 in the replicative senescent primary RPE cells treated with or without 1 μM IPI-504 for 24 hours.

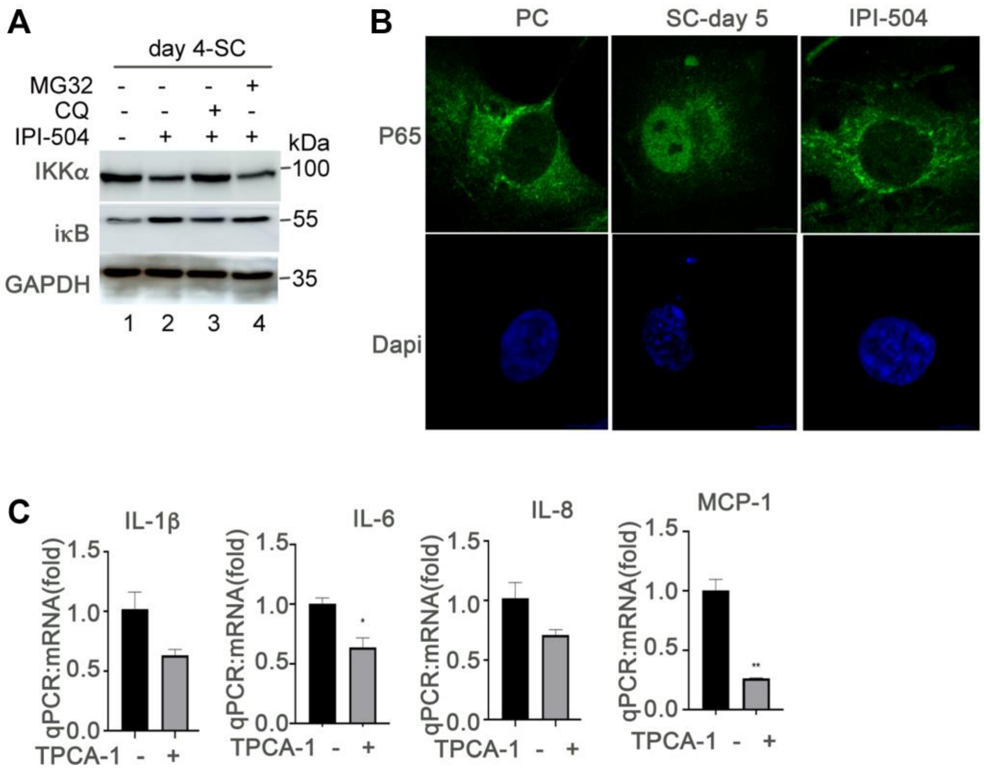

Supplementary Figure 5. IKKα-NF-κB pathway is associated with HSP90-regulated SASP in senescent RPE cells. (**A**) Immunoblot of IKKα, iκB and GAPDH proteins in day-4 senescent ARPE-19 cells treated with PBS (sham, lane 1), 5 μM IPI-504 (lane 2), 5 μM IPI-504 + 10 μM chloroquine (CQ:24 h, lane 3) and 5 μM IPI-504 + 10 μM MG132 (MG132:6 h, lane 4). (**B**) Immunofluorescence staining to determine the localization of P65 proteins in proliferating ARPE-19 cells (PC), day-5 senescent ARPE-19 cells (SC-day 5) and day-5 senescent ARPE-19 cells treated with 1 μM IPI-504 for 24 hours (IPI-504). The cell nucleus was stained with DAPI. (**C**) Quantitative PCR to determine the mRNA expression of IL-1β, IL-6, IL-8 and MCP-1 in replicative senescent primary monkey RPE cells (day-5) treated with DMSO (sham) or 1 μM TPCA-1 (IKKα/IKKβ inhibitor) for 24 hours. The results are mean ± SD (n = 4). The unpaired 2-tailed t- test was used for statistical analysis. $^{*}P < 0.01$, $^{**}P < 0.001$.

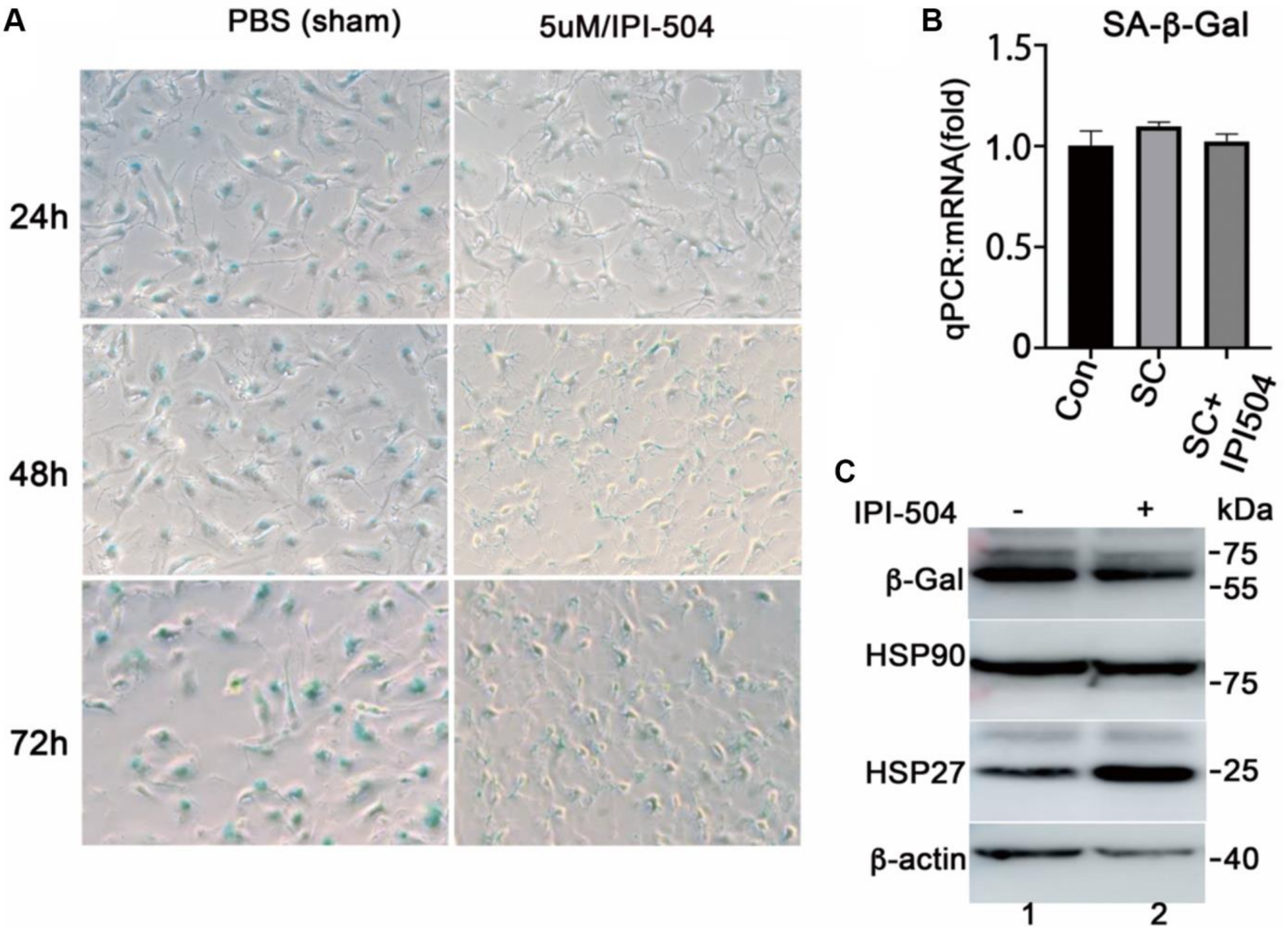

Supplementary Figure 6. IPI-504 inhibits SA-b-Gal protein expression and activity. (**A**) SA-b-Gal staining in the senescent ARPE-19 cells treated with sham (PBS) or 5 µM IPI-504. The day 2 recovery cells after 2 hour H_2O_2 treatment were incubated with complete media containing sham (PBS) or 5 µM IPI-504 for up to 72 hours. The cells were stained for SA-b-Gal. (**B**) quantitative PCR to determine mRNA level of SA-b-Gal in control ARPE-19 cells, (Con), day-4 senescent ARPE-19 cells (SC) and day-4 senescent ARPE-19 treated with 5 µM IPI-504 (SC + IPI-504) for 24 hours. (**C**) Immunoblot of b-Gal proteins expression in Hela cells treated with sham (PBS, lane 1) and 1 µM IPI-504 for 24 hours.

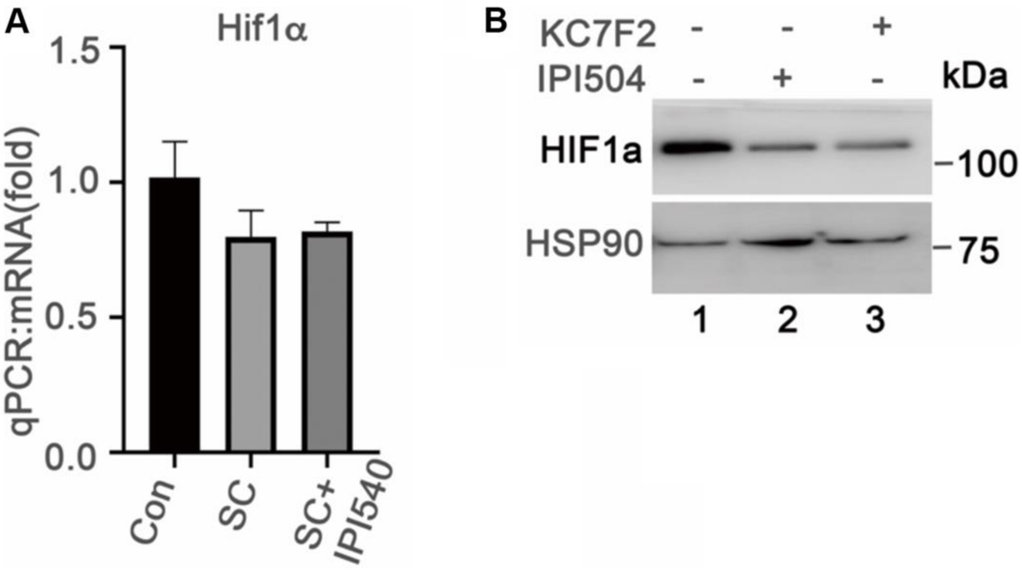

Supplementary Figure 7. The IPI-504 inhibits HIF1a protein expression in senescent ARPE-19 cells. (**A**) quantitative PCR to determine HIF1a mRNA in proliferating ARPE-19 cells (Con), day-4 senescent ARPE-19 cells (SC) and Day-4 senescent ARPE-19 cells (SC) treated with 1 µM IPI-504 for 24 hours (SC + IPI-504). (**B**) Immunoblot of HIF1a and HSP90a in day-4 senescent ARPE-19 cells treated with sham (PBS, lane 1), IPI-504 (lane 2) and KC7F2, an inhibitor of HIF1a (lane 3).

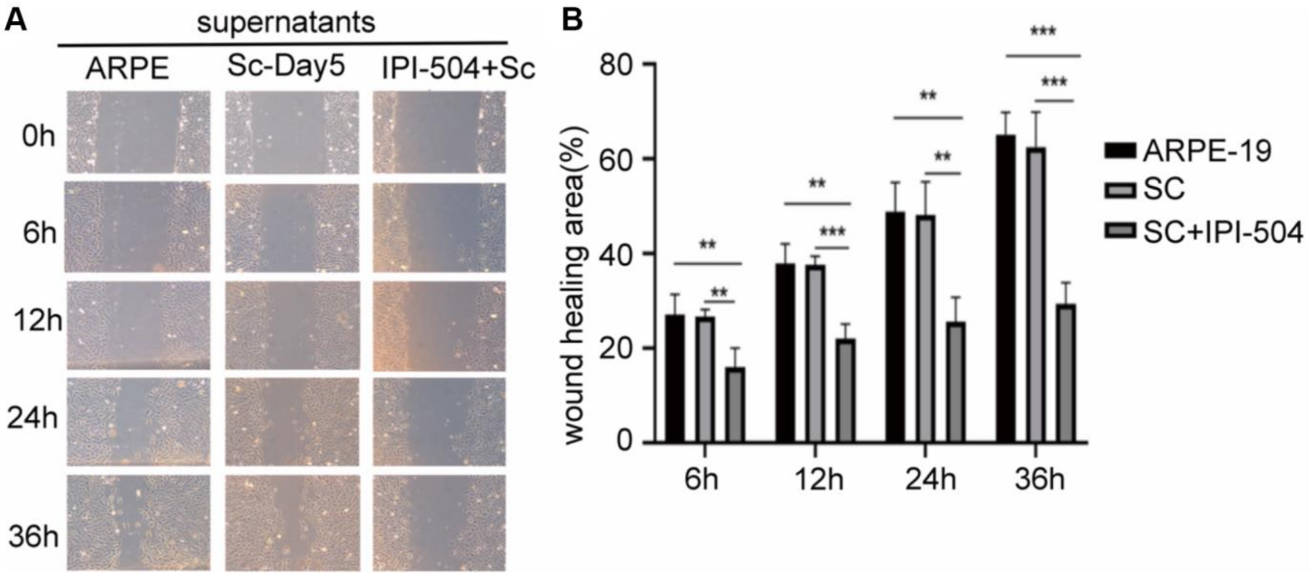

Supplementary Figure 8. IPI-504 inhibits senescent ARPE-19 mediated cell migration. (**A**) The wound-healing assay, the confluent ARPE-19 cells were scratched and incubated with conditional supernatants from proliferating ARPE-19 cells, Day-5 senescent ARPE-19 cells and Day-5 senescent ARPE-19 cells pretreated by 5 µM IPI-504 for 24 hours. (**B**) Quantitation of the area of wound closure in A in image J. The results were from three independent experiments. The unpaired 2-tailed *t*-test was used for statistical analysis. $^{**}P < 0.01$; $^{***}P < 0.001$.
